# Sex-specific obesity paradox and type 2 myocardial infarction in acute ischemic stroke (AIS) patients

**DOI:** 10.1186/s13293-026-00823-x

**Published:** 2026-01-16

**Authors:** Wan Wang, Man Huang, Wu-lin Li, Xiao-wei Du, Yue Li, Zhao-hui Lu, Bei-bei Sun, Yu-qing Mao, Xiao-ya Ma, Xiao Han, Xiao Wu, Hui Chen, Fei Wang

**Affiliations:** 1 Department of Neurology, Central Hospital Affiliated Shanghai University of Medicine & Health Sciences, No.1, Chengbei Rd, Jiading District, Shanghai, China; 2 Department of Nursing, Central Hospital Affiliated Shanghai University of Medicine & Health Sciences, Jiading District, Shanghai, China; 3 Department of Emergency and Critical Care Medicine, Central Hospital Affiliated Shanghai University of Medicine & Health Sciences, No.1, Chengbei Rd, Jiading District, Shanghai, China; 4 Department of Cardiology, Central Hospital Affiliated Shanghai University of Medicine & Health Sciences, Jiading District, Shanghai, China

**Keywords:** Obesity, Sex, Paradox, Acute ischemic stroke, Type 2 myocardial Infarction

## Abstract

**Background and aims:**

Obesity is usually linked to negative outcomes in many diseases; however, some acute critical conditions exhibit a phenomenon known as the obesity paradox. This investigation assessed sex-specific differences in type 2 myocardial infarction (T2MI), a condition caused by an imbalance between oxygen supply and demand in the myocardium and unrelated to atherosclerotic plaque rupture. Additionally, the study explored the implications of body mass index (BMI) in patients with acute ischemic stroke (AIS).

**Methods:**

AIS patients were consecutively enrolled at Jiading District Central Hospital affiliated Shanghai University of Medicine & Health Sciences, from October 1, 2017, to December 31, 2023. Participants were divided into four groups based on their BMI: underweight group, normal weight group, overweight group, and obesity group. The primary outcome of the study was the incidence of T2MI. We employed Cox regression analysis and Kaplan–Meier curves to examine the relationship between BMI and the occurrence of T2MI. Additionally, we performed a restricted cubic spline (RCS) analysis to evaluate the linearity of this relationship, utilizing an iterative algorithm to pinpoint inflection points. The subgroup forest plot displays how the four BMI groups vary across different layers.

**Results:**

The incidence of T2MI was 4.43%(131/2995) in AIS patients. After adjusting for potential confounding variables, the risk of T2MI was higher in the normal-weight group (HR, 2.11; 95% CI, 1.36–3.26; p < 0.001) compared to the obese group. In female patients, the risk of T2MI was higher in both the normal-weight group (HR, 3.47; 95% CI, 1.64–7.36; P = 0.001) and the underweight group (HR, 4.06; 95% CI, 1.44–11.44; P = 0.008) compared to the obese group; however, no such association was found in male patients. Furthermore, the RCS analysis confirmed a linear correlation between BMI and the risk of T2MI.

**Conclusions:**

The association between BMI and T2MI in AIS patients varied between genders. Obese female AIS patients had a lower risk of T2MI, a trend that was not mirrored in their male counterparts. These findings underscore the importance of considering sex-specific factors in understanding the complex relationship between obesity and T2MI in patients with AIS.

**Graphical Abstract:**

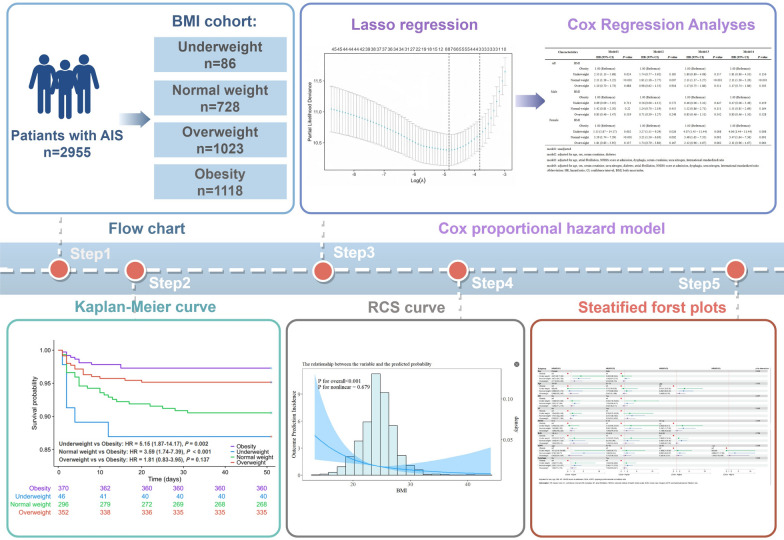

**Supplementary Information:**

The online version contains supplementary material available at 10.1186/s13293-026-00823-x.

## Abstract (plain language summary)

In AIS patients, obese individuals, especially females, showed a significantly lower risk of experiencing T2MI compared to their non-obese counterparts. After adjusting for potential confounding variables, we consistently observed that normal-weight patients were more likely to experience T2MI than those classified as obese. Normal-weight and underweight females had a higher risk of T2MI compared to obese females, but this difference was not seen in males. The study showed that an obesity paradox exists in female patients with AIS regarding the incidence of T2MI. This highlights the importance of considering gender-specific factors to understand the complex relationship between obesity and T2MI in AIS patients.

**Supplementary Information:**

The online version contains supplementary material available at 10.1186/s13293-026-00823-x.

## Highlights

- In AIS patients, females are at higher risk for T2MI than males.
- A sex-specific obesity paradox relates to the occurrence of T2MI in AIS patients. Female patients with normal-weight or underweight had a higher risk of T2MI than obese female patients, but no such difference was found among male patients.

**Supplementary Information:**

The online version contains supplementary material available at 10.1186/s13293-026-00823-x.

## Introduction

Obesity is considered a major global health issue [1], significantly increasing the risk of heart and brain-related diseases [2]. However, the "obesity paradox" shows that obese individuals can have better clinical outcomes in specific diseases [3]. Research findings on the relationship between obesity and cardiovascular events in patients are inconsistent. Patients face risks from metabolic disorders, yet the "obesity paradox" suggests a contrasting clinical phenomenon. Studies have established a strong association between obesity and metabolic disorders in patients with AIS [4, 5]. The presence of these disorders is linked to a higher risk of death [6, 7] and significantly increases the risk of cardiovascular events, such as myocardial infarction [8]. Meanwhile, higher BMI predicted the occurrence of the first coronary heart disease event [9, 10], suggesting a potentially increased risk of myocardial infarction.

However, some studies offer a contrasting view. A Korean study shows that among diabetic patients with stable conditions after acute myocardial infarction (AMI), the heart disease mortality rate and all-cause mortality rate in non-obese patients are significantly higher than in obese patients [11]. They found that among stroke patients with type 2 diabetes, the risk of major adverse cardiovascular events (MACE) is 13% lower in the overweight group (BMI 25–29.9 kg/m^2^) and 42% lower in the obese group (BMI ≥ 30 kg/m^2^) compared to the normal weight group [12]. Patients with obesity class I (BMI 30–34.9 kg/m^2^) have a lower incidence of perioperative myocardial infarction during non-cardiac surgery [13]. Among patients with severe peripheral artery disease, those with low BMI experienced a higher incidence of major adverse cardiovascular events (including myocardial infarction, or stroke) at 7.9%, compared to 4.1% in the normal BMI group (p = 0.003) [14]. These findings suggest a potential link between the obesity paradox and myocardial infarction.

Type 2 myocardial infarction (T2MI) results from myocardial necrosis caused by an imbalance between oxygen supply and demand. T2MI is a subtype of acute myocardial infarction (AMI) unrelated to coronary artery plaque damage [15]. The risk factors for T2MI include advanced age, diabetes, hypoxemia, severe hypertension, and hypotension [16], while the role of gender as a risk factor remains controversial [15, 17, 18]. AMI is relatively uncommon following acute stroke, occurring in 1.6% of stroke patients [19]. However, when it does occur, it significantly increases the risk of adverse outcomes. Moreover, even among survivors, it imposes a significant burden on families and society [20]. This finding suggests that T2MI occurrence in AIS patients should be taken seriously. Additionally, research indicates significant gender differences in the impact of obesity on clinical outcomes in patients undergoing heart surgery [21, 22]. This indicates that gender differences are closely associated with the obesity paradox. Therefore, this study aimed to investigate gender-specific differences in T2MI incidence by BMI among Chinese AIS patients. It also sought to determine whether the obesity paradox exists within each gender group.

## Methods

### Study design and population

This study was designed as a retrospective cohort study.

This retrospective study included consecutive patients who were diagnosed with AIS at Jiading District Central Hospital affiliated Shanghai University of Medicine & Health Sciences, from October 1, 2017, to December 31, 2023. Eligible participants met the predefined inclusion and exclusion criteria. The diagnostic standards for AIS in this research were based on the Chinese Guidelines for the Diagnosis and Treatment of AIS, referencing the 2014 edition prior to 2018 and the 2018 edition thereafter [23, 24]. The diagnostic criteria for AIS were: (1) sudden onset; (2) focal neurological deficits such as unilateral weakness or numbness in the face or limbs, or language impairments, possibly leading to severe neurological dysfunction; (3) imaging evidence of lesions or symptoms/signs lasting more than 24 h; (4) exclusion of non-vascular causes; (5) cerebral CT or MRI to rule out cerebral hemorrhage.

The criteria for diagnosing myocardial infarction were established according to the Universal Definition of Myocardial Infarction, 3rd and 4th editions [25, 26]. The details are delineated as follows: 1. A rise and/or fall in cTn values, with a minimum of one measurement surpassing the 99th percentile; 2. one or more clinical indicators of myocardial ischemia, including: (1) symptoms suggestive of acute myocardial ischemia; (2) new ischemic changes on electrocardiogram, such as ST-segment changes or new left bundle branch block; (3) pathological Q waves; (4) imaging evidence of new loss of viable myocardium or regional wall motion abnormalities consistent with ischemia; (5) definitive evidence obtained from coronary angiography.

The diagnostic standards for T2MI are as follows. Patients were categorized as "objective T2MI" if they presented with at least one objective clinical feature of acute myocardial ischemia **(Supplementary Table 1)**[27]. Patients with T2MI were further categorized as "subjective T2MI" when ischemic symptoms were the only criteria for diagnosis. Ischemic symptoms included retrosternal chest discomfort, dyspnea, palpitations, diaphoresis, lightheadedness, presyncope, syncope, upper abdominal pain, heartburn unrelated to meals, nausea, and vomiting [28]. When the diagnosis was unclear, a cardiovascular specialist and two physicians from the emergency and critical care department independently evaluated the case. A T2MI diagnosis required confirmation by at least two physicians.

The inclusion criteria are: (1) meeting the diagnostic standards for AIS; (2) being 18 years or older.

The exclusion criteria are: (1) confirmed cerebral or subarachnoid hemorrhage by brain CT or MRI; (2) transient ischemic attack (TIA); (3) non-vascular causes; (4) other types of stroke, such as moyamoya disease; (5) other conditions associated with elevated cTn levels, including Type 1 myocardial infarction, acute myocardial injury, and chronic myocardial injury; (6) treatment discontinuation; (7) incomplete information; and (8) pregnancy.

### Study methods

Gather baseline demographic information for all patients, including sex, age, BMI, and lifestyle factors such as drinking and smoking status.

The past medical history includes various conditions such as cerebral hemorrhage, cerebral infarction, hypertension, diabetes, myocardial infarction, atrial fibrillation, hyperlipidemia, senile dementia, mental illnesses, chronic obstructive pulmonary disease (COPD), family history of stroke, transient ischemic attack, and heart valve replacement surgery.

In this study, the dual antiplatelet therapy (DAPT) regimen consists of aspirin (100 mg once daily) combined with clopidogrel (75 mg once daily). This regimen is the standard for secondary prevention in patients with acute ischemic stroke in our clinical practice. It is used unless specific contraindications exist, such as high bleeding risk or allergy. If a loading dose is given, it consists of 300 mg of aspirin and 300 mg of clopidogrel. The standard duration of DAPT is 21 days [29].

The prior medication history includes antiplatelet therapy as monotherapy (aspirin 100 mg once daily) or dual antiplatelet therapy (DAPT, aspirin 100 mg plus clopidogrel 75 mg once daily). It also includes anticoagulants, hypolipidemic agents, hypoglycemic drugs, and antihypertensive medications.

Cerebral reperfusion therapy (CRT) involves two primary treatments: intravenous thrombolysis using alteplase and mechanical thrombectomy.

The types of stroke in patients were classified using the Oxfordshire Community Stroke Project (OCSP) and Trial of ORG 10172 in Acute Stroke Treatment (TOAST) classifications.

Stroke severity at admission was assessed using the modified Rankin Scale (mRS) and National Institutes of Health Stroke Scale (NIHSS) scores, along with factors such as dysphagia, mean arterial pressure (MAP), and pulse rate.

The laboratory parameters assessed included cardiac troponin (cTn), low-density lipoprotein, homocysteine, HbA1c, fasting blood glucose, serum creatinine, blood urea nitrogen, uric acid, and international normalized ratio (INR).

All results were from the first examination conducted at admission to the ward, based on treatments performed during the main period. The data have been meticulously processed and systematically organized within an Excel database.

The Body Mass Index (BMI) was calculated as weight (kg) divided by the square of height (m) [30]. According to the World Health Organization (WHO) and American Diabetes Association (ADA) criteria for Asians, patients were classified into four groups based on BMI at the time of AIS. These groups were defined as underweight (< 18.5 kg/m^2^), normal weight (18.5–22.99 kg/m^2^), overweight (23.0–24.99 kg/m^2^), and obese (≥ 25.0 kg/m^2^) [5].

Subsequently, the estimated glomerular filtration rate (eGFR) was calculated using the Chronic Kidney Disease Epidemiology Collaboration (CKD-EPI) equation to assess the risk of renal impairment. The equation is as follows: eGFR = 141 × [min(SCr/κ, 1)]^α × [max(SCr/κ, 1)]^(-1.209) × 0.993^age × (1.018 if female). Here, κ equals 0.7 for females and 0.9 for males. The functions min(SCr/κ, 1) and max(SCr/κ, 1) return the smaller and larger value of SCr/κ and 1, respectively [31].

The primary outcome of the study was the incidence of T2MI. Secondary outcomes included in-hospital all-cause mortality, 90-day all-cause mortality, and pneumonia rate. Additionally, we recorded the length of hospital stay, total hospitalization costs, drug costs, and NIHSS and MRS scores at discharge.

### Statistical methods

Data processing and analysis were performed using R version 4.3.3 (2024–02-29). Missing data were assessed for all variables. Variables with less than 20% missingness were handled by multiple imputation using the ‘mice’ R package. Variables with missingness ≥ 20% were excluded from the analysis. Continuous variables were expressed as means ± SDs or medians (P25, P75) and compared using Student’s t-test or Mann–Whitney U test. Categorical variables are presented as counts and percentages and analyzed by Pearson’s Chi-square test or Fisher’s exact test. P < 0.05 was considered statistically significant.

Survival analyses were conducted using Kaplan–Meier curves and log-rank tests to examine the effect of BMI on T2MI. The Bonferroni method was used to adjust for multiple pairwise comparisons among data groups. We used LASSO regression to select variables linked to the risk of T2MI. Covariate selection was informed by clinical experience and prior research. Core clinical variables, chosen based on clinical experience and prior studies, were included in the LASSO model to identify stable and interpretable covariates. Additionally, four regression models were used: Model 1 with no covariate adjustment; Model 2 adjusted for clinical factors and prior literature [16]; Model 3 adjusted for independent risk factors identified by Cox regression; and Model 4 adjusted for covariates from Models 2 and 3. Cox proportional hazards regression models were used to estimate the adjusted hazard ratio (aHR) of prognosis according to the BMI group. The multivariate Cox regression models included clinically important variables and variables with adjustment for different baseline characteristics. The proportional hazards assumption was evaluated using statistical and graphical methods based on scaled Schoenfeld residuals. To address any violations, stratified Cox models were applied, ensuring the reliability of the results.

We constructed Restricted Cubic Spline (RCS) curves to clarify the relationship between BMI and the risk of T2MI occurrence. Additionally, we used a iterative algorithm to identify inflection points of T2MI in the entire population and in male and female subsets. To explore differences between men and women, we performed BMI subgroup analyses separately for each sex. Hazard ratios (HRs) for T2MI occurrence were analyzed by BMI subgroups in males and females to assess BMI’s differential effects. Statistical significance was defined as p < 0.05.

## Results

### Descriptive analysis

**Fig. 1:**
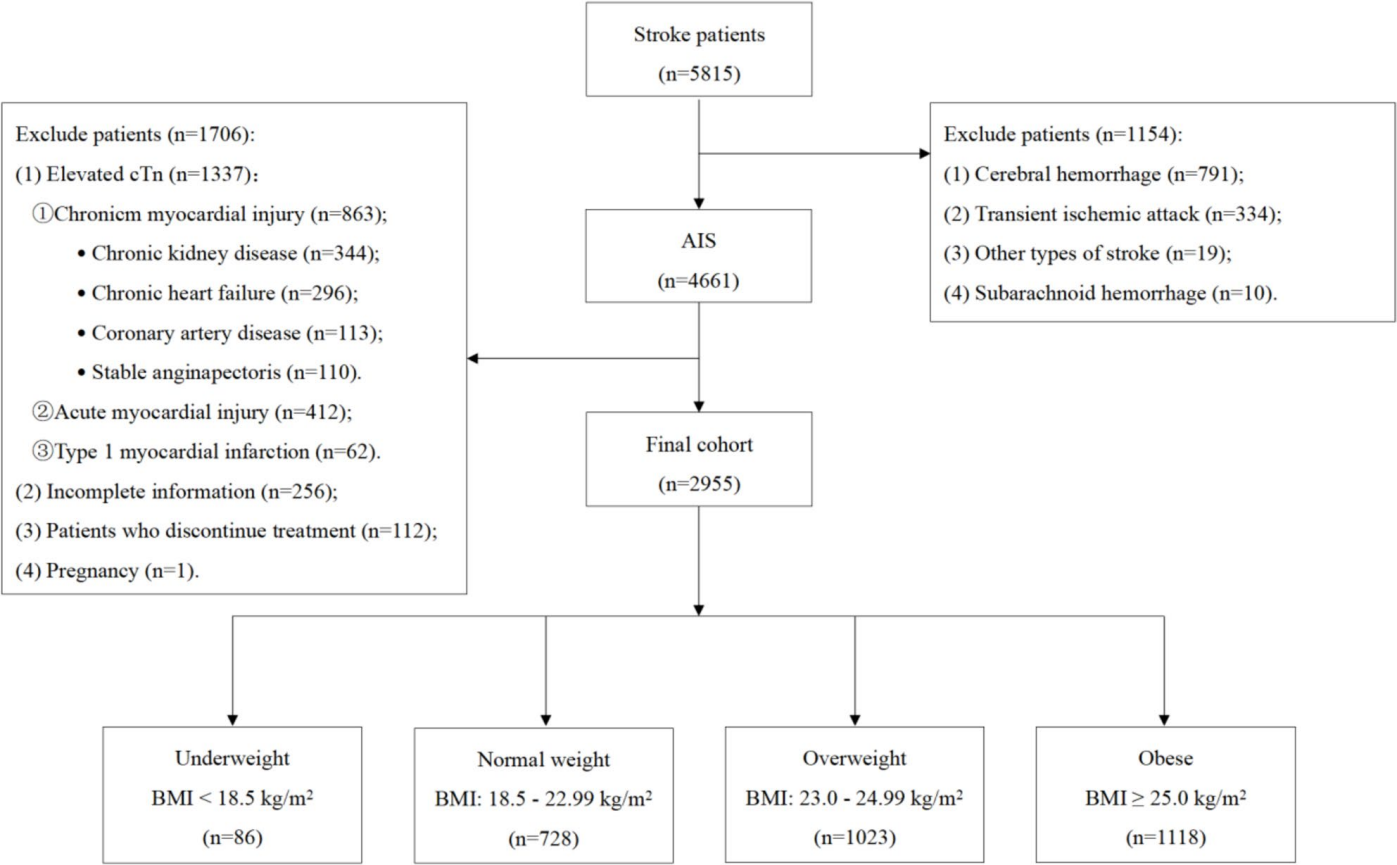
The study flowchart

A total of 4661 AIS patients were hospitalized between October 1, 2017, and December 31, 2023. Based on the established inclusion and exclusion criteria, 2955 patients were included in the analysis. The patient selection process is illustrated in Fig. 1. These patients were classified into four groups according to their BMI upon admission.

### Baseline characteristics

The average age of all AIS patients was 70.32 years (± 13.35), with females comprising 36.01% of the cohort (1064/2955). Among them, 71.84% (2123/2955) had hypertension, and 30.32% (896/2955) had diabetes. During hospitalization, 39.73% (1174/2955) of patients underwent single-drug antiplatelet therapy, whereas 53.16% (1571/2955) underwent dual antiplatelet therapy. Additionally, 43.15% (1275/2955) received hypolipidemic drug therapy, and 75.67% (2236/2955) received blood pressure medication. Regarding CRT therapy, intravenous thrombolysis with alteplase was administered to 15.13% (447 out of 2955) of patients, whereas arterial thrombectomy was performed on 1.70% (48 out of 2955). The average ages differed significantly among the weight groups (obese: 68.13 ± 13.99; overweight: 70.78 ± 12.67; normal weight: 72.27 ± 12.79; underweight: 76.97 ± 12.16), with all comparisons to the obese group showing P < 0.001. Furthermore, the percentage of females in these groups showed a significant increase: 33.09% in the obese group, 34.41% in the overweight group, 40.66% in the normal weight group, and 53.49% in the underweight group, with a P-value of less than 0.001. Significant declines were observed in the proportions of individuals with a drinking history (obese 20.57%, overweight 15.54%, normal weight 14.15%, underweight 6.98%; P < 0.001), hypolipidemic drug therapy during hospitalization (obese 45.62%, overweight 44.97%, normal weight 38.74%, underweight 26.74%; P < 0.001), and use of blood pressure medication (obese 79.43%, overweight 76.54%, normal weight 70.74%, underweight 58.14%; P < 0.001).

The incidence of T2MI in AIS was 4.43% (131/2955). Compared to the obese cohort, the incidence of T2MI was significantly higher in the overweight, normal weight, and underweight categories (3.31% vs. 3.62% vs. 6.87% vs. 8.14%, P < 0.001). No significant differences were found in the rates of pneumonia, in-hospital all-cause mortality, or 90-day all-cause mortality among the groups (see Table 1 for detailed statistics and comparisons). The median follow-up period for T2MI was 4 days (interquartile range 8 days), and for death, it was 19 days (interquartile range 39 days).

**Table 1:** Baseline and clinical prognostic characteristics in all patients

Variables	Total (n = 2955)	Underweight(n = 86)	Normal weight (n = 728)	Overweight (n = 1023)	Obesity (n = 1118)	*P*-value
Demographic data						
Female, n (%)	1064 (36.01)	46 (53.49)	296 (40.66)	352 (34.41)	370 (33.09)	< 0.001
Age (Mean ± SD, years)	70.32 ± 13.35	76.97 ± 12.16	72.27 ± 12.79	70.78 ± 12.67	68.13 ± 13.99	< 0.001
BMI, (Mean ± SD, kg/m^2)^	24.39 ± 2.97	17.29 ± 1.16	21.33 ± 1.16	24.12 ± 0.55	27.17 ± 2.08	< 0.001
OCSP, n (%)						0.013
TACI	326 (11.03)	12 (13.95)	82 (11.26)	113 (11.05)	119 (10.64)	
PACI	1763 (59.66)	62 (72.09)	449 (61.68)	583 (56.99)	669 (59.84)	
POCI	748 (25.31)	11 (12.79)	161 (22.12)	291 (28.45)	285 (25.49)	
LACI	118 (3.99)	1 (1.16)	36 (4.95)	36 (3.52)	45 (4.03)	
TOAST, n (%)						< 0.001
LAA	1177 (39.83)	31 (36.05)	271 (37.23)	418 (40.86)	457 (40.88)	
CE	375 (12.69)	19 (22.09)	90 (12.36)	137 (13.39)	129 (11.54)	
SAA	1311 (44.37)	29 (33.72)	340 (46.70)	449 (43.89)	493 (44.10)	
Other etiological types	40 (1.35)	3 (3.49)	18 (2.47)	8 (0.78)	11 (0.98)	
Unexplained	52 (1.76)	4 (4.65)	9 (1.24)	11 (1.08)	28 (2.50)	
Drinking, n (%)	498 (16.85)	6 (6.98)	103 (14.15)	159 (15.54)	230 (20.57)	< 0.001
Smoking, n (%)						0.234
Never smoked	2109 (71.37)	60 (69.77)	544 (74.73)	720 (70.38)	785 (70.21)	
Previously smoked, now quit	132 (4.47)	4 (4.65)	28 (3.85)	55 (5.38)	45 (4.03)	
Currently still smoking	714 (24.16)	22 (25.58)	156 (21.43)	248 (24.24)	288 (25.76)	
Past medical history, n (%)						
Cerebral hemorrhage	79 (2.67)	0 (0.00)	16 (2.20)	32 (3.13)	31 (2.77)	0.278
Cerebral infarction	570 (19.29)	15 (17.44)	121 (16.62)	234 (22.87)	200 (17.89)	0.004
Hypertension	2123 (71.84)	52 (60.47)	479 (65.80)	760 (74.29)	832 (74.42)	< 0.001
Diabetes	896 (30.32)	18 (20.93)	199 (27.34)	334 (32.65)	345 (30.86)	0.024
Myocardial infarction	16 (0.54)	0 (0.00)	2 (0.27)	6 (0.59)	8 (0.72)	0.648
Atrial fibrillation	429 (14.52)	19 (22.09)	101 (13.87)	149 (14.57)	160 (14.31)	0.235
Hyperlipoidemia	16 (0.54)	1 (1.16)	4 (0.55)	5 (0.49)	6 (0.54)	0.68
Senile dementia	33 (1.12)	1 (1.16)	8 (1.10)	14 (1.37)	10 (0.89)	0.779
Mental disorder	12 (0.41)	1 (1.16)	3 (0.41)	5 (0.49)	3 (0.27)	0.405
COPD	59 (2.00)	6 (6.98)	18 (2.47)	15 (1.47)	20 (1.79)	0.004
Spontaneous intracerebral hemorrhage	39 (1.32)	2 (2.33)	7 (0.96)	20 (1.96)	10 (0.89)	0.106
Family history of stroke	12 (0.41)	0 (0.00)	3 (0.41)	2 (0.20)	7 (0.63)	0.496
Transient ischemic attack	18 (0.61)	0 (0.00)	4 (0.55)	4 (0.39)	10 (0.89)	0.491
Heart valve replacement surgery	5 (0.17)	0 (0.00)	0 (0.00)	1 (0.10)	4 (0.36)	0.367
Previous medication history, n (%)						
Antiplatelet therapy						0.171
Non-antiplatelet	2561 (86.67)	73 (84.88)	646 (88.74)	874 (85.43)	968 (86.58)	
Single drug antiplatelet	362 (12.25)	13 (15.12)	78 (10.71)	132 (12.90)	139 (12.43)	
Duplex antiplatelet agglutination therapy	32 (1.08)	0 (0.00)	4 (0.55)	17 (1.66)	11 (0.98)	
Anticoagulant medication	46 (1.56)	1 (1.16)	8 (1.10)	13 (1.27)	24 (2.15)	0.244
Hypolipidemic drug therapy	287 (9.71)	9 (10.47)	59 (8.10)	100 (9.78)	119 (10.64)	0.346
Blood pressure medication	1622 (54.89)	39 (45.35)	364 (50.00)	587 (57.38)	632 (56.53)	0.003
Hypoglycemic medication	688 (23.28)	13 (15.12)	150 (20.60)	257 (25.12)	268 (23.97)	0.039
Hospital medication, n (%)						
Antiplatelet therapy						< 0.001
Non-antiplatelet	210 (7.11)	11 (12.79)	54 (7.42)	73 (7.14)	72 (6.44)	
Single drug antiplatelet	1174 (39.73)	50 (58.14)	294 (40.38)	401 (39.20)	429 (38.37)	
Duplex antiplatelet agglutination therapy	1571 (53.16)	25 (29.07)	380 (52.20)	549 (53.67)	617 (55.19)	
Anticoagulant medication	91 (3.08)	2 (2.33)	18 (2.47)	27 (2.64)	44 (3.94)	0.215
Hypolipidemic drug therapy	1275 (43.15)	23 (26.74)	282 (38.74)	460 (44.97)	510 (45.62)	< 0.001
Blood pressure medication	2236 (75.67)	50 (58.14)	515 (70.74)	783 (76.54)	888 (79.43)	< 0.001
Hypoglycemic medication	988 (33.43)	16 (18.60)	206 (28.30)	370 (36.17)	396 (35.42)	< 0.001
Cerebral vascular reperfusion therapy (CRT), n (%)						
Alteplase intravenous thrombolysis	447 (15.13)	30 (34.88)	143 (19.64)	112 (10.95)	162 (14.49)	< 0.001
Arterial embolectomy	48 (1.70)	2 (2.47)	17 (2.45)	9 (0.93)	20 (1.85)	0.1
Severity of illness						
MRS score at admission, M (IQR)	2 (1, 2)	2 (1,2)	2 (1,2)	2 (1,2)	1 (1,2)	< 0.001
NIHSS score at admission, M (IQR)	2 (1, 6)	3 (1,12)	2 (1,6)	2 (1,6)	2 (1,6)	0.015
Dysphagia, n (%)	430 (14.75)	21 (24.42)	107 (14.92)	131 (12.97)	171 (15.52)	0.024
MAP, (Mean ± SD, mmHg)	106.10 ± 14.56	101.68 ± 13.13	103.86 ± 13.73	105.65 ± 13.67	108.31 ± 15.62	< 0.001
Pulse, (Mean ± SD, Times/minute)	78.24 ± 14.93	80.65 ± 19.30	77.14 ± 15.53	77.99 ± 14.48	79.00 ± 14.52	0.025
Laboratory parameters						
Cardiac troponin (cTn), [M (IQR), ng/mL]	0.01 (0.01, 0.02)	0.01 (0.01,0.03)	0.01 (0.01,0.02)	0.01 (0.01,0.02)	0.01 (0.01,0.02)	0.044
Low-density lipoprotein, [M (IQR), mmol/L]	2.64 (2.03, 3.30)	2.30 (1.98,2.96)	2.57 (2.00,3.22)	2.65 (2.03,3.31)	2.70 (2.09,3.35)	0.007
Homocysteine, [M (IQR), umol/L]	15.10 (11.50, 20.70)	14.95 (11.50,20.58)	14.90 (11.30,20.20)	15.10 (11.60,20.30)	15.20 (11.70,21.22)	0.529
HbA1c, Mean ± SD	6.67 ± 1.71	6.37 ± 1.50	6.50 ± 1.63	6.72 ± 1.72	6.76 ± 1.76	0.003
Fasting blood glucose, (Mean ± SD, mmol/L)	6.73 ± 2.69	6.24 ± 2.29	6.47 ± 2.46	6.86 ± 2.87	6.83 ± 2.68	0.004
Serum creatinine, [M (IQR), umol/L]	73.00 (61.98, 87.10)	69.84 (62.35,82.90)	71.00 (61.21,86.10)	72.82 (61.53,86.35)	74.76 (62.60,89.00)	0.014
Blood urea nitrogen, [M (IQR), mmol/L]	5.20 (4.20, 6.40)	5.30 (4.32,6.80)	5.10 (4.20,6.40)	5.20 (4.20,6.40)	5.20 (4.20,6.40)	0.763
Uric acid, (Mean ± SD, umol/L)	331.10 ± 104.16	313.33 ± 110.11	316.78 ± 102.49	327.28 ± 100.93	345.31 ± 106.02	< 0.001
International normalized ratio, Mean ± SD	0.96 ± 0.14	0.96 ± 0.08	0.96 ± 0.10	0.96 ± 0.15	0.96 ± 0.15	0.998
clinical prognosis						
T2MI, n (%)	131 (4.43)	7 (8.14)	50 (6.87)	37 (3.62)	37 (3.31)	< 0.001
Pneumonia, n (%)	666 (22.54)	26 (30.23)	170 (23.35)	229 (22.39)	241 (21.56)	0.281
Died in hospital, n (%)	57 (1.93)	4 (4.65)	18 (2.47)	18 (1.76)	17 (1.52)	0.13
Died in 90 days, n (%)	107 (3.62)	7 (8.14)	30 (4.12)	33 (3.23)	37 (3.31)	0.097
Length of hospital stay, [M (IQR), days]	11.00 (8.00, 13.00)	11.00 (8.00,13.00)	11.00 (8.00,14.00)	10.00 (8.00,13.00)	11.00 (8.00,14.00)	0.456
NIHSS score at discharge, M (IQR)	2 (1, 5)	3 (1,9.75)	2 (1,5)	2(1,5)	2 (1,6)	0.042
MRS score at discharge, M (IQR)	2 (1, 3)	2(1,4)	2(1,3)	2 (1,3)	2 (1,3)	0.041
Total hospitalization expenses, [M (IQR), ten thousand yuan]	1.557(1.177, 2.123)	1.572(1.258,2.254)	1.564(1.174,2.169)	1.491 (1.144,2.003)	1.576 (1.217,2.181)	0.006
Total cost of medication, [M (IQR), ten thousand yuan]	0.729(0.454, 1.095)	0.723(0.534,1.140)	0.732(0.430,1.102)	0.712 (0.446,1.039)	0.748(0.481,1.133)	0.052

BMI, body mass index; OCSP, oxfordshire community stroke project; TACI, total anterior circulation infarcts; PACI, partial anterior circulation infarcts; POCI, posterior circulation infarcts; LACI, lacunar infarcts; TOAST, trial of org 10,172 in acute stroke treatment; LAA, large-artery atherosclerosis; CE, cardioembolism; SAA, small-artery occlusion lacunar; COPD, chronic obstructive pulmonary disease; mRs, modified rankin scale; NIHSS, national institute of health stroke scale; MAP, mean arterial pressure; HbA1c, glycated hemoglobin; T2MI, type 2 myocardial infarction

Among females, the incidence of T2MI was recorded at 5.73% (61/1064). The incidence of T2MI was markedly higher in the overweight (4.83%), normal weight (9.46%), and underweight (13.04%) groups compared to the obese group (2.70%, P < 0.001), as illustrated in Table 2. However, this disparity was not observed among males, as shown in Supplementary Table 2.

**Table 2:** Baseline and clinical prognostic characteristics in female patients

Variables	Female (n = 1064)	Underweight(n = 46)	Normal weight (n = 296)	Overweight (n = 352)	Obesity(n = 370)	*P*-value
Demographic data						
Age (Mean ± SD, years)	74.94 ± 12.33	79.89 ± 12.13	75.52 ± 12.94	75.20 ± 11.39	73.60 ± 12.55	0.005
BMI, (Mean ± SD, kg/m^2)^	24.06 ± 3.14	17.30 ± 1.24	21.26 ± 1.16	24.03 ± 0.60	27.17 ± 2.24	< 0.001
OCSP, n (%)						0.521
TACI	143 (13.44)	8 (17.39)	40 (13.51)	46 (13.07)	49 (13.24)	
PACI	625 (58.74)	31 (67.39)	176 (59.46)	204 (57.95)	214 (57.84)	
POCI	249 (23.40)	6 (13.04)	63 (21.28)	91 (25.85)	89 (24.05)	
LACI	47 (4.42)	1 (2.17)	17 (5.74)	11 (3.12)	18 (4.86)	
TOAST, n (%)						0.088
LAA	399 (37.50)	15 (32.61)	114 (38.51)	117 (33.24)	153 (41.35)	
CE	184 (17.29)	12 (26.09)	50 (16.89)	71 (20.17)	51 (13.78)	
SAA	450 (42.29)	17 (36.96)	122 (41.22)	154 (43.75)	157 (42.43)	
Other etiological types	12 (1.13)	0 (0.00)	7 (2.36)	3 (0.85)	2 (0.54)	
Unexplained	19 (1.79)	2 (4.35)	3 (1.01)	7 (1.99)	7 (1.89)	
Drinking, n (%)	22 (2.07)	0 (0.00)	1 (0.34)	10 (2.84)	11 (2.97)	0.049
Smoking, n (%)						0.032
Never smoked	1043 (98.03)	45 (97.83)	293 (98.99)	345 (98.01)	360 (97.30)	
Previously smoked, now quit	5 (0.47)	0 (0.00)	3 (1.01)	0 (0.00)	2 (0.54)	
Currently still smoking	16 (1.50)	1 (2.17)	0 (0.00)	7 (1.99)	8 (2.16)	
Past medical history, n (%)						
Cerebral hemorrhage	24 (2.26)	0 (0.00)	2 (0.68)	12 (3.41)	10 (2.70)	0.076
Cerebral infarction	196 (18.42)	7 (15.22)	44 (14.86)	69 (19.60)	76 (20.54)	0.237
Hypertension	807 (75.85)	32 (69.57)	216 (72.97)	265 (75.28)	294 (79.46)	0.17
Diabetes	304 (28.57)	11 (23.91)	75 (25.34)	103 (29.26)	115 (31.08)	0.358
Myocardial infarction	2 (0.19)	0 (0.00)	0 (0.00)	2 (0.57)	0 (0.00)	0.271
Atrial fibrillation	223 (20.96)	13 (28.26)	60 (20.27)	81 (23.01)	69 (18.65)	0.302
Hyperlipoidemia	6 (0.56)	1 (2.17)	1 (0.34)	1 (0.28)	3 (0.81)	0.285
Senile dementia	20 (1.88)	1 (2.17)	7 (2.36)	7 (1.99)	5 (1.35)	0.806
Mental disorder	7 (0.66)	0 (0.00)	2 (0.68)	3 (0.85)	2 (0.54)	0.925
COPD	10 (0.94)	1 (2.17)	5 (1.69)	1 (0.28)	3 (0.81)	0.167
Spontaneous intracerebral hemorrhage	12 (1.13)	1 (2.17)	3 (1.01)	7 (1.99)	1 (0.27)	0.089
Family history of stroke	5 (0.47)	0 (0.00)	0 (0.00)	1 (0.28)	4 (1.08)	0.277
Transient ischemic attack	4 (0.38)	0 (0.00)	1 (0.34)	1 (0.28)	2 (0.54)	1
Heart valve replacement surgery	3 (0.28)	0 (0.00)	0 (0.00)	1 (0.28)	2 (0.54)	0.807
Previous medication history, n (%)						
Antiplatelet therapy						0.726
Non-antiplatelet	931 (87.50)	40 (86.96)	258 (87.16)	315 (89.49)	318 (85.95)	
Single drug antiplatelet	126 (11.84)	6 (13.04)	37 (12.50)	34 (9.66)	49 (13.24)	
Duplex antiplatelet agglutination therapy	7 (0.66)	0 (0.00)	1 (0.34)	3 (0.85)	3 (0.81)	
Anticoagulant medication	21 (1.97)	0 (0.00)	3 (1.01)	8 (2.27)	10 (2.70)	0.319
Hypolipidemic drug therapy	99 (9.30)	4 (8.70)	24 (8.11)	29 (8.24)	42 (11.35)	0.418
Blood pressure medication	640 (60.15)	23 (50.00)	172 (58.11)	213 (60.51)	232 (62.70)	0.319
Hypoglycemic medication	241 (22.65)	9 (19.57)	59 (19.93)	82 (23.30)	91 (24.59)	0.497
Hospital medication, n (%)						
Antiplatelet therapy						0.266
Non-antiplatelet	96 (9.02)	6 (13.04)	25 (8.45)	35 (9.94)	30 (8.11)	
Single drug antiplatelet	496 (46.62)	23 (50.00)	145 (48.99)	145 (41.19)	183 (49.46)	
Duplex antiplatelet agglutination therapy	472 (44.36)	17 (36.96)	126 (42.57)	172 (48.86)	157 (42.43)	
Anticoagulant medication	43 (4.04)	0 (0.00)	6 (2.03)	14 (3.98)	23 (6.22)	0.023
Hypolipidemic drug therapy	479 (45.02)	13 (28.26)	124 (41.89)	174 (49.43)	168 (45.41)	0.027
Blood pressure medication	804 (75.56)	28 (60.87)	204 (68.92)	270 (76.70)	302 (81.62)	< 0.001
Hypoglycemic medication	332 (31.20)	10 (21.74)	75 (25.34)	117 (33.24)	130 (35.14)	0.019
Cerebral vascular reperfusion therapy (CRT), n (%)						
Alteplase intravenous thrombolysis	183 (17.20)	20 (43.48)	70 (23.65)	43 (12.22)	50 (13.51)	< 0.001
Arterial embolectomy	21 (2.05)	0 (0.00)	10 (3.56)	3 (0.89)	8 (2.21)	0.093
Severity of illness						
MRS score at admission, M (IQR)	2 (1, 2)	2 (1,2)	2 (1,2)	2 (1,2)	1 (1,2)	0.168
NIHSS score at admission, M (IQR)	3 (1, 9)	3.5 (1.25,12)	3 (1,8)	3 (1,8)	3 (1,9.75)	0.267
Dysphagia, n (%)	221 (20.97)	12 (26.09)	63 (21.43)	60 (17.14)	86 (23.63)	0.144
MAP, (Mean ± SD, mmHg)	105.51 ± 14.24	103.78 ± 12.43	103.46 ± 14.06	106.13 ± 14.26	106.78 ± 14.43	0.015
Pulse, (Mean ± SD, Times/minute)	78.00 (69.00, 87.00)	79.50 (71.00,94.00)	78.00 (68.00,86.00)	80.00 (68.75,88.00)	78.00 (70.00,86.00)	0.49
Laboratory parameters						
Cardiac troponin (cTn), [M (IQR), ng/mL]	0.01 (0.01, 0.02)	0.02 (0.01,0.04)	0.01 (0.01,0.02)	0.01 (0.01,0.02)	0.01 (0.01,0.02)	0.061
Low-density lipoprotein, [M (IQR), mmol/L]	2.71 (2.11, 3.41)	2.38 (2.12,2.94)	2.63 (2.09,3.34)	2.85 (2.19,3.45)	2.75 (2.11,3.48)	0.047
Homocysteine, [M (IQR), umol/L]	13.90 (10.50, 19.20)	15.85 (11.20,20.58)	13.60 (10.20,19.70)	14.00 (10.85,18.55)	13.80 (10.33,18.78)	0.524
HbA1c, Mean ± SD	6.60 ± 1.59	6.28 ± 1.09	6.33 ± 1.34	6.76 ± 1.68	6.71 ± 1.71	0.001
Fasting blood glucose, (Mean ± SD, mmol/L)	6.81 ± 2.68	6.32 ± 1.82	6.49 ± 2.35	6.98 ± 2.74	6.96 ± 2.91	0.037
Serum creatinine, [M (IQR), umol/L]	64.14 (54.83, 79.03)	66.96 (53.19,78.94)	64.05 (53.48,78.09)	64.06 (54.95,79.10)	64.10 (55.29,79.94)	0.86
Blood urea nitrogen, [M (IQR), mmol/L]	5.00 (4.10, 6.30)	5.15 (4.32,6.20)	5.00 (4.10,6.40)	5.00 (4.10,6.40)	5.10 (4.10,6.20)	0.973
Uric acid, (Mean ± SD, umol/L)	310.09 ± 106.69	295.50 ± 120.44	301.14 ± 106.65	306.41 ± 101.67	322.57 ± 108.79	0.038
International normalized ratio, Mean ± SD	0.96 ± 0.16	0.95 ± 0.07	0.96 ± 0.08	0.96 ± 0.15	0.98 ± 0.22	0.433
clinical prognosis						
T2MI, n (%)	61 (5.73)	6 (13.04)	28 (9.46)	17 (4.83)	10 (2.70)	< 0.001
Pneumonia, n (%)	295 (27.73)	12 (26.09)	84 (28.38)	97 (27.56)	102 (27.57)	0.987
Died in hospital, n (%)	30 (2.82)	2 (4.35)	14 (4.73)	6 (1.70)	8 (2.16)	0.089
Died in 90 days, n (%)	52 (4.89)	5 (10.87)	19 (6.42)	14 (3.98)	14 (3.78)	0.085
Length of hospital stay, [M (IQR), days]	11.00 (9.00, 14.00)	11.00 (8.00,13.00)	11.00 (8.00,15.00)	11.00 (9.00,14.00)	11.00 (9.00,16.00)	0.052
NIHSS score at discharge, M (IQR)	3 (1, 8)	3(1,10.5)	2 (1,7)	3 (1,8)	3(1,8)	0.26
MRS score at discharge, M (IQR)	2(1, 4)	3 (1,4)	2 (1,4)	2 (1,4)	2 (1,4)	0.336
Total hospitalization expenses, [M (IQR), ten thousand yuan]	1.602(1.201, 2.227)	1.532 (1.22,2.26)	1.658(1.193,2.288)	1.553 (1.172,2.004)	1.631 (1.268,2.403)	0.043
Total cost of medication, [M (IQR), ten thousand yuan]	0.775 (0.481, 1.177)	0.796 (0.534,1.204)	0.767 (0.450,1.152)	0.738 (0.467,1.074)	0.824 (0.499,1.291)	0.116

Abbreviation: BMI, body mass index; OCSP, oxfordshire community stroke project; TACI, total anterior circulation infarcts; PACI, partial anterior circulation infarcts; POCI, posterior circulation infarcts; LACI, lacunar infarcts; TOAST, trial of org 10,172 in acute stroke treatment; LAA, large-artery atherosclerosis; CE, cardioembolism; SAA, small-artery occlusion lacunar; COPD, chronic obstructive pulmonary disease; mRs, modified rankin scale; NIHSS, national institute of health stroke scale; MAP, mean arterial pressure; HbA1c, glycated hemoglobin; T2MI, type 2 myocardial infarction

### Risk analysis of T2MI

**Fig. 2:**
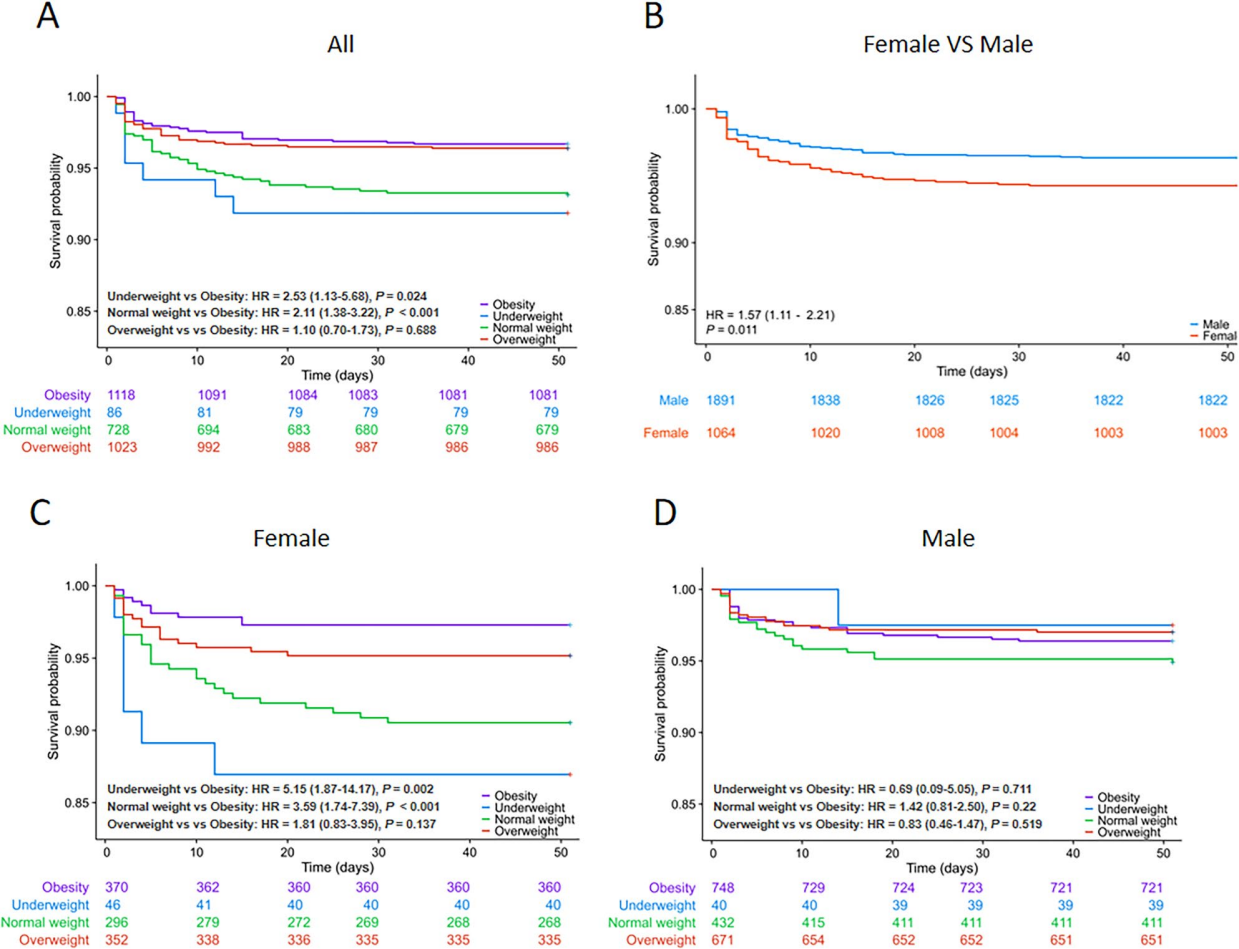
Kaplan–Meier curves for T2MI occurrence. (**A**, All patients; **B**, Different genders; **C**, Female patients; **D**, Male patients)

The cumulative risk assessment of T2MI among AIS patients showed that the normal weight and underweight cohorts had a significantly higher risk of T2MI compared to the obese cohort (p < 0.001 and p = 0.024, respectively) (Fig. 2A). Additionally, female patients exhibited a higher risk of T2MI compared to male patients (HR 1.57, 95% CI 1.11–2.21, P < 0.001) (Fig. 2B). A further subgroup analysis indicated that among females, the cumulative risk of T2MI was significantly higher in the normal weight group (p < 0.001) and the underweight group (p = 0.002) compared to the obese group (Fig. 2C). Conversely, no significant difference in cumulative risk of T2MI was observed among male participants (Fig. 2D).

### Screening of risk factors for T2MI

In the overall cohort, the T2MI group had a significantly higher proportion of underweight and normal weight individuals compared to the Non-T2MI group (Table 3). Similar findings appeared among females, as shown in Supplementary Table 3; however, this higher proportion of underweight and normal weight individuals was not observed among males (Supplementary Table 4).

**Table 3:** Baseline Characteristics Of AIS Patients With T2MI occurrence in all patients

Variables	Total (n = 2955)	Non-T2MI (n = 2824)	T2MI (n = 131)	*P-value*	Univariate COX regression analysis	
					HR (95%CI)	*P*-value
Demographic data						
Female	1064 (36.01)	1003 (35.52)	61 (46.56)	0.01	1.57 (1.11 ~ 2.21)	0.011
Age	70.32 ± 13.35	69.97 ± 13.27	77.88 ± 12.72	< 0.001	1.05 (1.04 ~ 1.07)	< 0.001
BMI				< 0.001		
Obesity	1118 (37.83)	1081 (38.28)	37 (28.24)		1.00 (Reference)	
Underweight	86 (2.91)	79 (2.80)	7 (5.34)		2.53 (1.13 ~ 5.68)	0.024
Normal weight	728 (24.64)	678 (24.01)	50 (38.17)		2.11 (1.38 ~ 3.22)	< 0.001
Overweight	1023 (34.62)	986 (34.92)	37 (28.24)		1.10 (0.70 ~ 1.73)	0.688
OCSP				0.006		
TACI	326 (11.03)	303 (10.73)	23 (17.56)		1.00 (Reference)	
PACI	1763 (59.66)	1678 (59.42)	85 (64.89)		0.68 (0.43 ~ 1.08)	0.103
POCI	748 (25.31)	729 (25.81)	19 (14.50)		0.35 (0.19 ~ 0.65)	< 0.001
LACI	118 (3.99)	114 (4.04)	4 (3.05)		0.48 (0.16 ~ 1.38)	0.17
TOAST				< 0.001		
LAA	1177 (39.83)	1134 (40.16)	43 (32.82)		1.00 (Reference)	
CE	375 (12.69)	323 (11.44)	52 (39.69)		3.98 (2.66 ~ 5.96)	< 0.001
SAA	1311 (44.37)	1279 (45.29)	32 (24.43)		0.67 (0.42 ~ 1.05)	0.082
Other etiological types	40 (1.35)	38 (1.35)	2 (1.53)		1.38 (0.33 ~ 5.68)	0.658
Unexplained	52 (1.76)	50 (1.77)	2 (1.53)		1.06 (0.26 ~ 4.38)	0.935
Drinking	498 (16.85)	484 (17.14)	14 (10.69)	0.054	0.58 (0.34 ~ 1.02)	0.058
Smoking				0.306		
Never smoked	2109 (71.37)	2009 (71.14)	100 (76.34)		1.00 (Reference)	
Previously smoked, now quit	132 (4.47)	129 (4.57)	3 (2.29)		0.47 (0.15 ~ 1.49)	0.199
Currently still smoking	714 (24.16)	686 (24.29)	28 (21.37)		0.83 (0.54 ~ 1.26)	0.369
Past medical history						
Cerebral hemorrhage	79 (2.67)	77 (2.73)	2 (1.53)	0.579	0.56 (0.14 ~ 2.25)	0.411
Cerebral infarction	570 (19.29)	537 (19.02)	33 (25.19)	0.08	1.41 (0.95 ~ 2.10)	0.086
Hypertension	2123 (71.84)	2016 (71.39)	107 (81.68)	0.01	1.76 (1.13 ~ 2.74)	0.012
Diabetes	896 (30.32)	855 (30.28)	41 (31.30)	0.804	1.05 (0.72 ~ 1.51)	0.815
Myocardial infarction	16 (0.54)	15 (0.53)	1 (0.76)	0.517	1.44 (0.20 ~ 10.30)	0.716
Atrial fibrillation	429 (14.52)	380 (13.46)	49 (37.40)	< 0.001	3.65 (2.56 ~ 5.20)	< 0.001
Hyperlipoidemia	16 (0.54)	14 (0.50)	2 (1.53)	0.156	2.91 (0.72 ~ 11.74)	0.134
Senile dementia	33 (1.12)	32 (1.13)	1 (0.76)	1	0.68 (0.10 ~ 4.87)	0.701
Mental disorder	12 (0.41)	11 (0.39)	1 (0.76)	0.42	1.95 (0.27 ~ 13.93)	0.507
COPD	59 (2.00)	58 (2.05)	1 (0.76)	0.476	0.37 (0.05 ~ 2.65)	0.323
Spontaneous intracerebral hemorrhage	39 (1.32)	37 (1.31)	2 (1.53)	1	1.17 (0.29 ~ 4.72)	0.829
Family history of stroke	12 (0.41)	11 (0.39)	1 (0.76)	0.42	1.89 (0.26 ~ 13.53)	0.525
Transient ischemic attack	18 (0.61)	17 (0.60)	1 (0.76)	0.559	1.27 (0.18 ~ 9.11)	0.81
Heart valve replacement surgery	5 (0.17)	5 (0.18)	0 (0.00)	1	0.00 (0.00 ~ Inf)	0.993
Previous medication history						
Antiplatelet therapy				0.256		
Non-antiplatelet	2561 (86.67)	2453 (86.86)	108 (82.44)		1.00 (Reference)	
Single drug antiplatelet	362 (12.25)	340 (12.04)	22 (16.79)		1.45 (0.92 ~ 2.30)	0.111
Duplex antiplatelet agglutination therapy	32 (1.08)	31 (1.10)	1 (0.76)		0.74 (0.10 ~ 5.28)	0.761
Anticoagulant medication	46 (1.56)	40 (1.42)	6 (4.58)	0.012	3.17 (1.40 ~ 7.20)	0.006
Hypolipidemic drug therapy	287 (9.71)	269 (9.53)	18 (13.74)	0.111	1.50 (0.91 ~ 2.47)	0.11
Blood pressure medication	1622 (54.89)	1539 (54.50)	83 (63.36)	0.046	1.43 (1.01 ~ 2.04)	0.048
Hypoglycemic medication	688 (23.28)	659 (23.34)	29 (22.14)	0.751	0.93 (0.62 ~ 1.41)	0.746
Hospital medication						
Antiplatelet therapy				< 0.001		
Non-antiplatelet	210 (7.11)	187 (6.62)	23 (17.56)		1.00 (Reference)	
Single drug antiplatelet	1174 (39.73)	1101 (38.99)	73 (55.73)		0.55 (0.35 ~ 0.89)	0.014
Duplex antiplatelet agglutination therapy	1571 (53.16)	1536 (54.39)	35 (26.72)		0.20 (0.12 ~ 0.33)	< 0.001
Anticoagulant medication	91 (3.08)	82 (2.90)	9 (6.87)	0.021	2.40 (1.22 ~ 4.72)	0.011
Hypolipidemic drug therapy	1275 (43.15)	1235 (43.73)	40 (30.53)	0.003	0.57 (0.40 ~ 0.83)	0.003
Blood pressure medication	2236 (75.67)	2155 (76.31)	81 (61.83)	< 0.001	0.51 (0.36 ~ 0.73)	< 0.001
Hypoglycemic medication	988 (33.43)	956 (33.85)	32 (24.43)	0.025	0.64 (0.43 ~ 0.95)	0.027
Cerebral vascular reperfusion therapy (CRT)						
Alteplase intravenous thrombolysis	447 (15.13)	422 (14.94)	25 (19.08)	0.196	1.34 (0.87 ~ 2.07)	0.19
Arterial embolectomy	48 (1.70)	42 (1.56)	6 (4.69)	0.02	2.95 (1.30 ~ 6.70)	0.01
Severity of illness						
MRS score at admission	2 (1, 2)	2 (1, 2)	2 (1, 3)	0.125	1.24 (1.09 ~ 1.40)	< 0.001
NIHSS score at admission	2 (1, 6)	2 (1, 6)	11 (3, 17)	< 0.001	1.09 (1.08 ~ 1.11)	< 0.001
Dysphagia	430 (14.75)	359 (12.89)	71 (54.62)	< 0.001	7.34 (5.19 ~ 10.36)	< 0.001
MAP	106.10 ± 14.56	106.24 ± 14.37	103.05 ± 18.06	0.048	0.98 (0.97 ~ 0.99)	0.014
Pulse	78.24 ± 14.93	77.98 ± 14.76	83.97 ± 17.36	< 0.001	1.02 (1.01 ~ 1.03)	< 0.001
Laboratory parameters						
Cardiac troponin (cTn)	0.01 (0.01, 0.02)	0.01 (0.01, 0.02)	0.18 (0.06, 0.48)	< 0.001	1.62 (1.51 ~ 1.74)	< 0.001
Low-density lipoprotein	2.64 (2.03, 3.30)	2.65 (2.04, 3.30)	2.39 (1.77, 3.11)	0.018	0.82 (0.68 ~ 0.99)	0.045
Homocysteine	15.10 (11.50, 20.70)	15.00 (11.50, 20.40)	18.10 (12.83, 25.93)	< 0.001	1.01 (1.01 ~ 1.02)	0.016
HbA1c	6.67 ± 1.71	6.68 ± 1.71	6.52 ± 1.71	0.322	0.95 (0.85 ~ 1.06)	0.324
Fasting blood glucose	6.73 ± 2.69	6.71 ± 2.67	7.29 ± 3.10	0.015	1.07 (1.01 ~ 1.13)	0.014
Serum creatinine	73.00 (61.98, 87.10)	72.56 (61.75, 86.20)	92.16 (69.99, 116.63)	< 0.001	1.01 (1.01 ~ 1.01)	< 0.001
Blood urea nitrogen	5.20 (4.20, 6.40)	5.10 (4.20, 6.30)	6.90 (5.35, 10.10)	< 0.001	1.21 (1.17 ~ 1.26)	< 0.001
Uric acid	331.10 ± 104.16	329.59 ± 101.58	363.65 ± 146.18	0.009	1.01 (1.01 ~ 1.01)	< 0.001
International normalized ratio	0.96 ± 0.14	0.96 ± 0.14	1.02 ± 0.15	< 0.001	2.78 (1.84 ~ 4.20)	< 0.001
clinical prognosis						
Pneumonia,	666 (22.54)	582 (20.61)	84 (64.12)	< 0.001	6.39 (4.47 ~ 9.13)	< 0.001
Died in hospital	57 (1.93)	34 (1.20)	23 (17.56)	< 0.001	12.48 (7.95 ~ 19.60)	< 0.001
Died in 90 days	107 (3.62)	75 (2.66)	32 (24.43)	< 0.001	9.53 (6.40 ~ 14.20)	< 0.001
Length of hospital stay	11 (8, 13)	10 (8, 13)	15 (10, 24)	< 0.001	1.06 (1.05 ~ 1.07)	< 0.001
NIHSS score at discharge	2 (1, 5)	2 (1, 5)	10 (3, 16)	< 0.001	1.11 (1.09 ~ 1.13)	< 0.001
MRS score at discharge	2 (1, 3)	2(1, 3)	4 (3, 5)	< 0.001	1.77 (1.58 ~ 1.98)	< 0.001
Total hospitalization expenses	1.557 (1.178, 2.123)	1.532 (1.171, 2.070)	2.458 (1.720, 4.003)	< 0.001	1.01 (1.01 ~ 1.01)	0.007
Total cost of medication	0.729 (0.454, 1.095)	0.720 (0.450, 1.071)	1.104 (0.700, 1.769)	< 0.001	1.01 (1.01 ~ 1.01)	< 0.001

HR, hazard ratio; CI, confidence interval; Abbreviation: BMI, body mass index; OCSP, oxfordshire community stroke project; TACI, total anterior circulation infarcts; PACI, partial anterior circulation infarcts; POCI, posterior circulation infarcts; LACI, lacunar infarcts; TOAST, trial of org 10,172 in acute stroke treatment; LAA, large-artery atherosclerosis; CE, cardioembolism; SAA, small-artery occlusion lacunar; COPD, chronic obstructive pulmonary disease; mRs, modified rankin scale; NIHSS, national institute of health stroke scale; MAP, mean arterial pressure; HbA1c, glycated hemoglobin; T2MI, type 2 myocardial infarction

### Multivariate analysis of the occurrence of T2MI

**Fig. 3:**
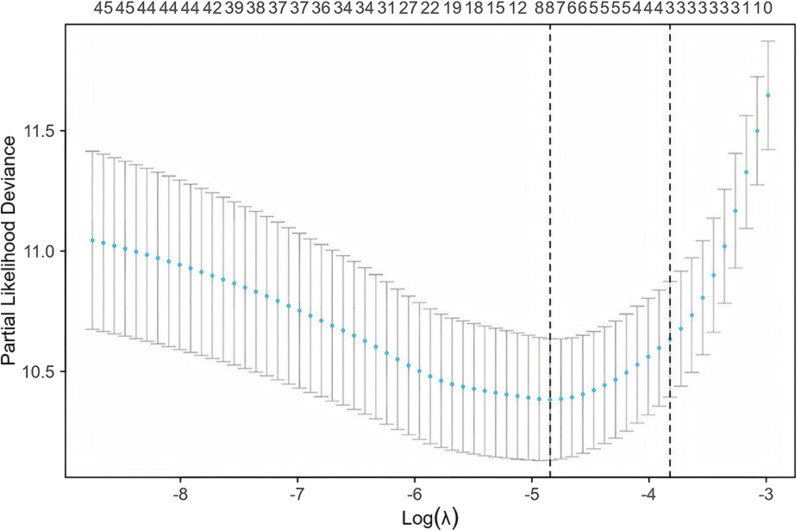
Lasso regression analysis to identify risk factors of T2MI

We used Lasso regression analysis (see Fig. 3) to identify risk factors associated with T2MI incidence. Ultimately, we identified eight relevant factors: BMI, age, history of atrial fibrillation, NIHSS score at admission, dysphagia, serum creatinine levels, urea nitrogen, and INR. These factors are summarized in Table 4.

**Table 4:** Univariate and multivariate Cox regression analysis of T2MI occurrence in all patients

Variables	Univariate analysis		Multivariate analysis	
	HR (95%CI)	*P*-value	HR (95%CI)	*P*-value
Model 1				
BMI(continuous variable)	0.88 (0.83 ~ 0.93)	< 0.001	0.91 (0.85 ~ 0.96)	< 0.001
Age	1.05 (1.04 ~ 1.07)	< 0.001	1.01 (1.00 ~ 1.03)	0.155
Atrial fibrillation	3.65 (2.56 ~ 5.20)	< 0.001	1.49 (0.99 ~ 2.25)	0.054
NIHSS score at admission	1.09 (1.08 ~ 1.11)	< 0.001	1.03 (1.01 ~ 1.06)	0.014
Dysphagia	7.34 (5.19 ~ 10.36)	< 0.001	2.89 (1.74 ~ 4.81)	< 0.001
Serum creatinine	1.01 (1.01 ~ 1.01)	< 0.001	1.00 (1.00 ~ 1.00)	0.296
Blood urea nitrogen	1.21 (1.17 ~ 1.26)	< 0.001	1.11 (1.05 ~ 1.18)	< 0.001
International normalized ratio	2.78 (1.84 ~ 4.20)	< 0.001	1.45 (0.72 ~ 2.96)	0.3
Model 2				
BMI 4 groups				
Obesity	1.00 (Reference)		1.00 (Reference)	
Underweight	2.53 (1.13 ~ 5.68)	0.024	1.80 (0.80 ~ 4.08)	0.157
Normal weight	2.11 (1.38 ~ 3.22)	< 0.001	2.11 (1.37 ~ 3.27)	< 0.001
Overweight	1.10 (0.70 ~ 1.73)	0.688	1.17 (0.73 ~ 1.86)	0.511
Age	1.05 (1.04 ~ 1.07)	< 0.001	1.01 (1.00 ~ 1.03)	0.15
Atrial fibrillation	3.65 (2.56 ~ 5.20)	< 0.001	1.53 (1.01 ~ 2.30)	0.042
NIHSS score at admission	1.09 (1.08 ~ 1.11)	< 0.001	1.03 (1.01 ~ 1.06)	0.012
Dysphagia	7.34 (5.19 ~ 10.36)	< 0.001	2.92 (1.76 ~ 4.85)	< 0.001
Serum creatinine	1.01 (1.01 ~ 1.01)	< 0.001	1.00 (1.00 ~ 1.00)	0.251
Blood urea nitrogen	1.21 (1.17 ~ 1.26)	< 0.001	1.11 (1.05 ~ 1.17)	< 0.001
International normalized ratio	2.78 (1.84 ~ 4.20)	< 0.001	1.48 (0.71 ~ 3.07)	0.296

HR, hazard ratio; CI, confidence interval; BMI, body mass index

Among patients with AIS, multivariate Cox regression analysis revealed that high NIHSS score at admission (HR 1.03 (1.01–1.06), P = 0.014), dysphagia (HR 2.89 (1.74–4.81), P < 0.001), and elevated blood urea nitrogen levels (HR 1.11 (1.05–1.18), P < 0.001) were associated with increased risk of T2MI after adjustment for relevant factors. A high BMI reduces the risk of T2MI (HR 0.91 (0.85–0.96), P < 0.001) (Model 1, Table 4). Compared to the obese group, those with normal BMI had a significantly higher risk of T2MI (HR 2.11 (1.37–3.27), P < 0.001) (Model 2, Table 4). Similar results were found among females (Supplementary Table 5), but this disparity was not observed among males (Supplementary Table 6).

### Association between BMI and risk of T2MI

To examine the association between BMI and T2MI in AIS patients, we used four Cox regression models. Initially, in the entire cohort, both the normal weight group (HR 2.11 (1.38–3.22), P < 0.001) and the underweight group (HR 2.53 (1.13–5.68), P = 0.024) showed a higher risk of T2MI than the obese group. A similar trend was observed in females, in which both the normal weight group (HR 3.59 (1.74–7.39), P < 0.001) and the underweight group (HR 5.15 (1.87–14.17), P = 0.002) showed a higher risk of T2MI than the obese group. No significant relationship between BMI and T2MI was observed in males. Detailed results can be found in Model 1 of Table 5.

**Table 5:** Cox regression analyses forT2MI occurrence of BMI groups

Characteristics		Model 1		Model 2		Model 3		Model 4	
		HR (95% CI)	*P*-value	HR (95% CI)	*P*-value	HR (95% CI)	*P*-value	HR (95% CI)	*P*-value
All	BMI								
	Obesity	1.00 (Reference)		1.00 (Reference)		1.00 (Reference)		1.00 (Reference)	
	Underweight	2.53 (1.13 ~ 5.68)	0.024	1.74 (0.77 ~ 3.92)	0.185	1.80 (0.80 ~ 4.08)	0.157	1.81 (0.80 ~ 4.10)	0.156
	Normal weight	2.11 (1.38 ~ 3.22)	< 0.001	1.81 (1.18 ~ 2.77)	0.007	2.11 (1.37 ~ 3.27)	< 0.001	2.11 (1.36 ~ 3.26)	< 0.001
	Overweight	1.10 (0.70 ~ 1.73)	0.688	0.98 (0.62 ~ 1.55)	0.918	1.17 (0.73 ~ 1.86)	0.511	1.17 (0.74 ~ 1.86)	0.503
Male	BMI								
	Obesity	1.00 (Reference)		1.00 (Reference)		1.00 (Reference)		1.00 (Reference)	
	Underweight	0.69 (0.09 ~ 5.05)	0.711	0.56 (0.08 ~ 4.15)	0.572	0.46 (0.06 ~ 3.41)	0.447	0.47 (0.06 ~ 3.49)	0.459
	Normal weight	1.42 (0.81 ~ 2.50)	0.22	1.24 (0.70 ~ 2.19)	0.455	1.52 (0.86 ~ 2.71)	0.151	1.51 (0.85 ~ 2.69)	0.164
	Overweight	0.83 (0.46 ~ 1.47)	0.519	0.71 (0.39 ~ 1.27)	0.248	0.83 (0.46 ~ 1.51)	0.542	0.83 (0.46 ~ 1.50)	0.528
Female	BMI								
	Obesity	1.00 (Reference)		1.00 (Reference)		1.00 (Reference)		1.00 (Reference)	
	Underweight	5.15 (1.87 ~ 14.17)	0.002	3.27 (1.15 ~ 9.29)	0.026	4.07 (1.45 ~ 11.44)	0.008	4.06 (1.44 ~ 11.44)	0.008
	Normal weight	3.59 (1.74 ~ 7.39)	< 0.001	3.21 (1.56 ~ 6.63)	0.002	3.48 (1.65 ~ 7.35)	0.001	3.47 (1.64 ~ 7.36)	0.001
	Overweight	1.81 (0.83 ~ 3.95)	0.137	1.74 (0.79 ~ 3.80)	0.167	2.12 (0.96 ~ 4.67)	0.062	2.11 (0.96 ~ 4.67)	0.064

model1: unadjusted

model2: adjusted for age, sex, serum creatinine, diabetes

model3: adjusted for age, atrial fibrillation, NHISS score at admission, dysphagia, serum creatinine, urea nitrogen, International normalized ratio

model4: adjusted for age, sex, serum creatinine, urea nitrogen, diabetes, atrial fibrillation, NHISS score at admission, dysphagia,, International normalized ratio

HR, hazard ratio; CI, confidence interval; BMI, body mass index

After adjusting for gender, age, diabetes history, and serum creatinine levels, normal weight AIS patients had a significantly higher risk of T2MI compared to obese patients. The hazard ratio was 1.81 (1.18–2.77), with a p-value of 0.007. In females, both the normal weight and underweight groups have a significantly higher risk of T2MI compared to the obese group. The normal weight group has a hazard ratio of 3.21 (1.56–6.63) and a p-value of 0.002, while the underweight group has a hazard ratio of 3.27 (1.15–9.29) and a p-value of 0.026. In contrast, no significant association was found in males. For more detailed information, please refer to Model 2 in Table 5.

Further analysis found that compared to the obese cohort, the normal weight group had a significantly increased risk of T2MI (HR 2.11 [1.37–3.27], P < 0.001). This result was derived after adjusting for age, atrial fibrillation history, NIHSS score at admission, dysphagia, serum creatinine, urea nitrogen, and INR. In females, the risk of T2MI was significantly higher in both normal weight and underweight groups (HR 3.48 [1.65–7.35], P = 0.001; HR 4.07 [1.45–11.44], P = 0.008, respectively). In contrast, no significant association was found in males. Detailed results can be found in Model 3 of Table 5.

Ultimately, the normal weight group showed a significantly higher risk of T2MI (HR 2.11 (1.36–3.26), P < 0.001) compared to the obese cohort. This result remained significant after adjusting for factors such as gender, age, diabetes history, serum creatinine levels, atrial fibrillation history, NIHSS score at admission, dysphagia, urea nitrogen, and INR. Among females, the risk of T2MI remained elevated in both the normal weight group (HR 3.47 (1.64–7.36), P = 0.001) and the underweight group (HR 4.06 (1.44–11.44), P = 0.008). However, there was no significant relationship between BMI and T2MI in males. Detailed results are presented in Model 4 of Table 5.

### Detection of linear relationship between BMI and T2MI

**Fig. 4:**
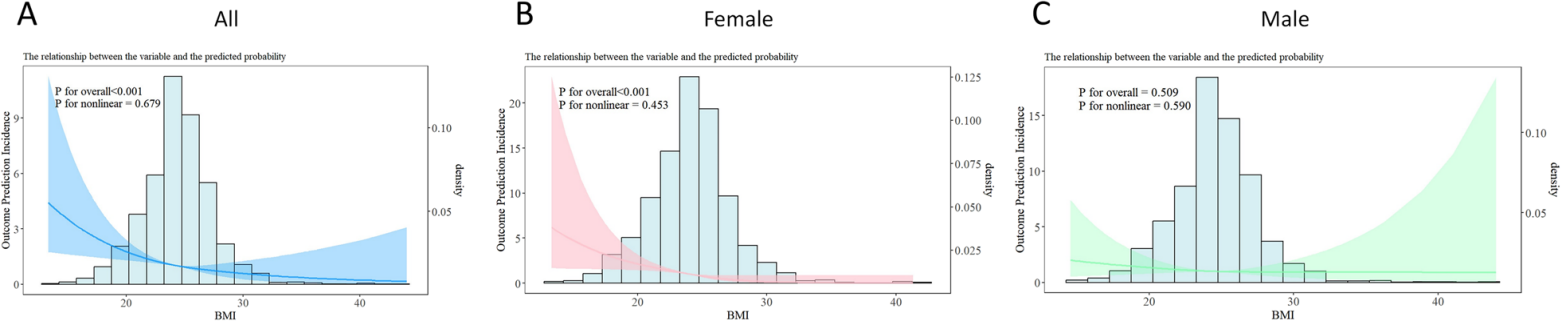
The RCS curve of BMI in relation to the incidence of T2MI. **A**, All patients. **B**, Female patients. **C**, Male patient

To examine the nonlinear relationship between BMI and the risk of T2MI, we employed a two-stage analytical approach. First, we applied restricted cubic splines (RCS) within a Cox proportional hazards regression to model BMI. This allowed visual examination of the nonlinear dose–response association with the outcome. All models were adjusted for covariates listed in Model 4 of Table 3. Subsequently, based on the nonlinear pattern suggested by the RCS curve, we applied a piecewise regression model to precisely estimate the potential inflection point (threshold) where the effect of BMI changes. The inflection point was identified using a maximum likelihood estimation (MLE) method via a grid search algorithm. The statistical necessity of introducing this threshold was formally tested using a likelihood ratio test, comparing the piecewise model against a standard Cox model with a linear term for BMI. In the overall cohort, each one-unit increase in BMI was associated with an 11.2% reduction in the risk of type 2 myocardial infarction (hazard ratio 0.888 [95% CI, 0.837–0.941], p < 0.001) (Fig. 4A). In females, each unit increase in BMI was linked to an 18.4% decrease in the risk of T2MI (hazard ratio 0.816 (0.75–0.888), p < 0.001) (Fig. 4B). However, the analysis did not reveal a significant association between BMI and the risk of type 2 myocardial infarction in males (Fig. 4C).

A two-stage Cox proportional hazards model was applied to explore the threshold effect of BMI on T2MI risk in AIS patients. In the entire cohort, the inflection point for BMI associated with T2MI was found to be 25.888. When BMI was below 25.888, each one-unit increase was linked to a 13.2% reduction in T2MI risk (hazard ratio 0.868 [95% CI, 0.811–0.929], p < 0.001). However, when BMI exceeds 25.888, the association between BMI and T2MI risk was not statistically significant. For females, the BMI inflection point is approximately 27.64. When BMI is below 27.64, each unit increase in BMI corresponds to a 16.8% reduction in the risk of T2MI (HR 0.832 (0.762–0.907), p < 0.001). However, when BMI exceeds 27.64, there is no significant relationship between BMI and the risk of T2MI. Although the piecewise fitting model did not significantly outperform the standard linear regression model (P for likelihood ratio test > 0.05), it offers clinically relevant insights. By introducing inflection points, this model clarifies the relationship between BMI and T2MI risk across different intervals (Table 6).

**Table 6:** Threshold effect analysis of BMI index on T2MI occurrence in AIS patients

Outcome	The effect size, 95%CI	*P*-value
All		
Model 1 Fitting model by standard linear regression	0.888(0.837–0.941)	< 0.001
Model 2 Fitting model by two-piecewise linear regression		
Inflection	25.888	
< 25.888	0.868(0.811–0.929)	< 0.001
> 25.888	0.989(0.83–1.178)	0.898
P for likelihood ratio test		0.255
Male		
Model 1 Fitting model by standard linear regression	0.957(0.879–1.042)	0.309
Model 2 Fitting model by two-piecewise linear regression		
Inflection	19.841	
< 19.841	1.477(0.675–3.231)	0.329
> 19.841	0.924(0.833–1.025)	0.134
P for likelihood ratio test		0.191
Female		
Model 1 Fitting model by standard linear regression	0.816(0.75–0.888)	< 0.001
Model 2 Fitting model by two-piecewise linear regression		
Inflection	27.64	
< 27.64	0.832(0.762–0.907)	< 0.001
> 27.64	0 (0-lnf)	0.987
P for likelihood ratio test		0.100

CI, confidence interval

### Subgroup stratified analysis of T2MI

**Fig. 5:**
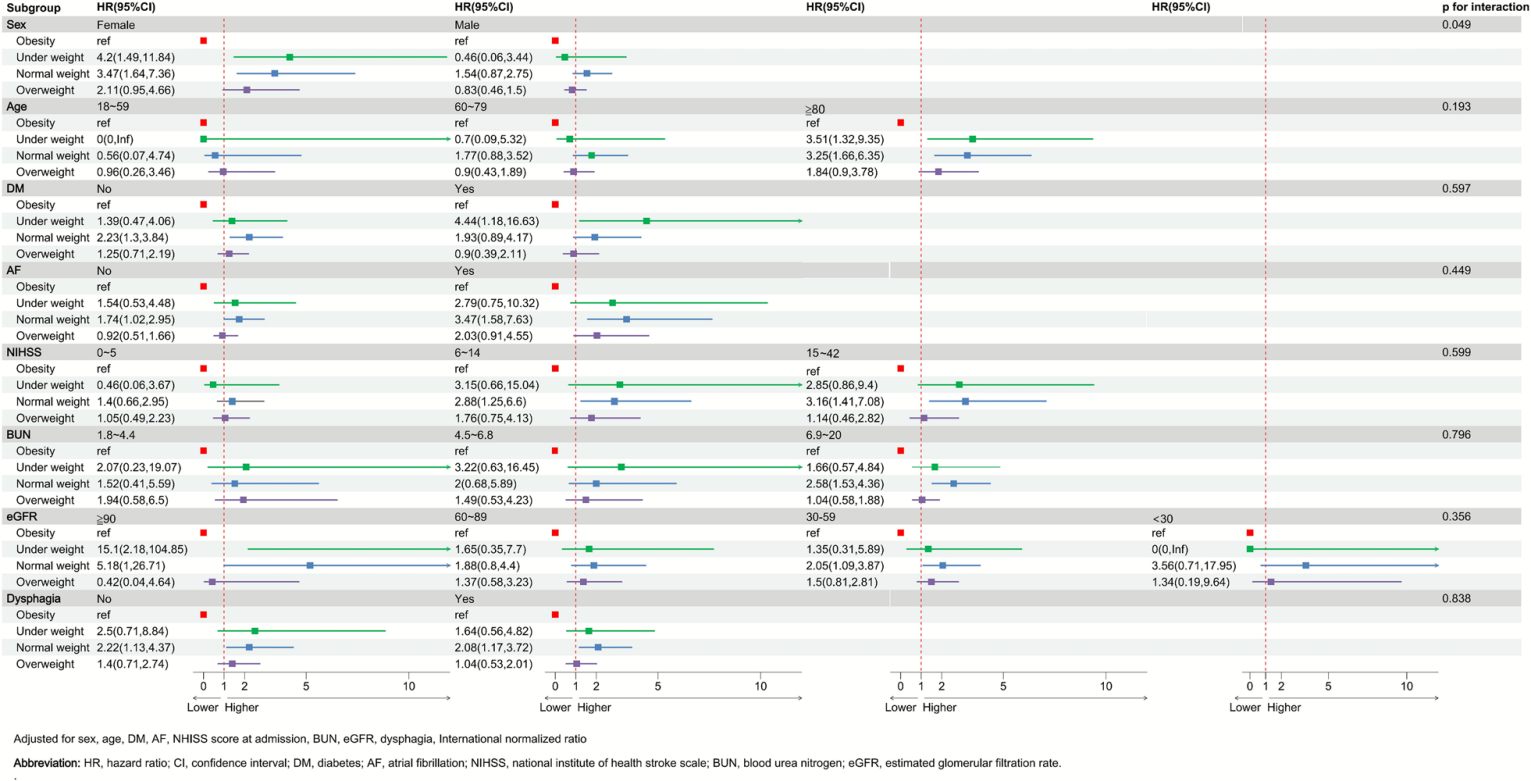
Subgroup analysis for risk of T2MI of BMI groups.

To explore the relationship between BMI categories and T2MI in different subgroups (Fig. 5), we performed stratified analyses based on age [32], diabetes mellitus, atrial fibrillation, NIHSS score at admission [33], dysphagia, eGFR [34], and BUN (tertiles). We applied adjustments similar to those used in Model 4 from Table 5.

Among female patients, the risk of T2MI was significantly higher in the underweight (HR: 4.2 [1.49–11.84]) and normal weight (HR: 3.47 [1.64–7.36]) groups than in the obese group. However, no significant differences were observed across weight categories in male patients. Patients in the underweight group who were aged ≥ 80 years, had diabetes mellitus, or an eGFR ≥ 90 showed a higher risk of T2MI compared with the obese group. Similarly, patients in the normal weight group who were aged ≥ 80 years, without diabetes, with or without atrial fibrillation, with an NIHSS score at admission between 6 and 42, a BUN level between 6.9 and 20, or an eGFR between 30 and 59 or ≥ 90, regardless of dysphagia status, also had a higher risk of T2MI compared with the obese group.

Furthermore, we observed a significant interaction between BMI and gender (P = 0.049), whereas no similar interactions were detected for other covariates. This indicates that gender significantly influences the relationship between BMI and T2MI occurrence.

### Sensitivity analysis—E-value analysis

E-value sensitivity analysis is performed to assess the impact of unmeasured confounding on the association between BMI and T2MI. For non-overweight vs. obesity (HR = 2.45, 95% CI: 1.55–3.86), the E-value for the point estimate was 4.33. For overweight vs. obesity (HR = 1.29, 95% CI: 0.79–2.09), the E-value was 1.90, as detailed in Table 7. Therefore, these results confirm the robustness of the core finding that Non-overweight AIS patients have a higher T2MI risk than obese patients.

**Table 7:** E-value Analysis for Unmeasured Confounding

Comparison	HR	95% CI	E-value for Point Estimate	E-value for CI Limit	Interpretation
Non-overweight vs Obesity	2.45	1.55–3.86	4.33	2.47	Robust to unmeasured confounding (E-value = 4.33)
Overweight vs Obesity	1.29	0.79–2.09	1.90		Potentially explainable by confounding (E-value = 1.9)

E-value analysis based on manual calculation using standard formulas for rare outcomes. Event rate in this study: 4.44%. E-value represents the minimum strength of association that an unmeasured confounder would need to have with both exposure and outcome to explain away the observed effect

## Discussion

This is the first study to investigate gender-based disparities in BMI and the incidence of T2MI in AIS, highlighting the obesity paradox in female patients. This finding emphasizes the importance of including sex-specific variables in understanding the complex relationship between obesity and T2MI prevalence in AIS.

Over one-third of the global population is classified as overweight or obese, and this trend is on the rise [35]. Moreover, obesity significantly increases the risk of chronic conditions, including depression, type 2 diabetes, cardiovascular diseases, and some cancers, while also raising mortality rates [36]. To address this urgent issue, the Chinese government launched the "Year of Weight Management" initiative in June 2024 [37]. Despite the significant impact of obesity on public health, research has produced inconsistent findings regarding its influence on stroke outcomes [38, 39]. Some studies have reported an obesity paradox in ischemic stroke patients, suggesting that obesity may result in improved functional recovery [40], lower mortality rates [41], a reduced likelihood of stroke recurrence [42], and fewer hospital readmissions [43]. Although studies indicate that a higher BMI is associated with better survival in AIS patients [35, 44], this study found no association between BMI and in-hospital or 90-day all-cause mortality. Several factors may explain this discrepancy: (1) The current study included a lower percentage of females than Liu’s study [30] (36.0% vs. 45.1%). Additionally, our study showed a higher proportion of obese individuals compared to the previous study [45] (37.83% vs. 22.8%), but a lower proportion of overweight individuals (34.62% vs. 41.6%). (2) Obese individuals often have additional health issues, such as hypertension and diabetes. Increased awareness of obesity’s harmful effects and advancements in national health policies have led to medical interventions that improve the management of comorbidities, potentially reducing obesity’s impact on mortality rates [46].

In patients with AIS, the incidence of Major Adverse Cardiovascular Events (MACE) was 55% higher in those with a BMI less than 18.5 kg/m^2^ compared to the normal-weight group (HR = 1.55, 95% CI: 1.01–2.38) [12]. Our study found that T2MI occurs more often in normal-weight patients (18.5–22.99 kg/m^2^) than in obese patients. BMI showed a significant inverse linear relationship with the incidence of T2MI. A significant association between BMI and T2MI was found when BMI was below 25.888; however, no correlation was observed when BMI exceeded this value. Further analysis by gender revealed that BMI showed no significant correlation with T2MI risk in male AIS patients. In contrast, among female patients with a BMI below 27.64, the risk of T2MI decreased as BMI increased. These findings suggest that clinicians should pay particular attention to monitoring the BMI of AIS patients, especially female patients.

This finding reinforces the concept of the obesity paradox. The mechanism behind this phenomenon can be explained as follows. (1) Obesity is a chronic metabolic disorder [47]. After a stroke, physiological stress can increase metabolic catabolism, which may cause nutritional risks. Research shows that the stroke-induced stress response involves neuroendocrine autonomic activation, release of pro-inflammatory cytokines, increased oxygen radical production, and systemic hormonal changes. Together, these factors create an overall catabolic state. In contrast, overweight or obese patients have greater energy reserves that may help counteract this excessive catabolism, leading to more favorable clinical outcomes and prognosis [35]. (2) Excessive body fat is the primary characteristic of obesity. Adipose tissue secretes pro-inflammatory cytokines, including TNF-α, IL-6, and MCP-1, which place the body in a state of chronic inflammation [35, 48, 49]. Individuals with obesity endure prolonged low-grade inflammation and develop inflammatory tolerance, which enables them to balance inflammatory responses [49, 50]. This mechanism plays a pivotal role throughout the various stages of atherosclerosis [51] and may contribute to improving myocardial ischemia in patients with AIS. Furthermore, soluble tumour necrosis factor receptors secreted by adipose tissue mitigate the detrimental effects of tumour necrosis factor-α on myocardial tissue [47]. This finding offers an explanation for the protective role of adipose tissue following acute myocardial infarction. (3) Moreover, adipose tissue secretion can reduce extracellular matrix fibrosis and macrophage infiltration and activation, while enhancing vascularization. These effects may protect the cardiovascular system. However, the precise mechanisms behind these protective actions require further investigation [52].

This study found that T2MI occurred in 4.43% of AIS cases. The right insular cortex is a key neuroanatomical region involved in stroke-related cardiac syndrome. This region plays a vital role in regulating cardiac function as part of the central autonomic nervous system. Specifically, damage to the right insular cortex reduces parasympathetic nerve activity. This reduction increases sympathetic nerve function, which consequently leads to acute myocardial injury [53].

Furthermore, females had a significantly higher risk of T2MI than males (HR 1.57 [1.11–2.21], P < 0.001). Among females with AIS, those who were normal weight or underweight showed a greater risk of T2MI compared to obese patients. This pattern was not observed in males, indicating a gender disparity in the "obesity paradox" related to T2MI in AIS. While the specific mechanisms behind this discrepancy remain unclear, research suggests several contributing factors. (1), individuals with obesity often exhibit higher lean body mass [54, 55]. Overall, women have approximately 15 to 20 kg less lean body mass than men, and they exhibit about a 36% higher total peripheral resistance. Research indicates that differences in lean body mass explain gender variations in cardiac output and peak exercise capacity among healthy men and women. Specifically, the lower lean body mass in females is a key factor influencing vasodilatory response, as individuals with greater lean body mass tend to exhibit stronger vasodilation. This results in a greater reduction in afterload during exercise, increased left ventricular end-diastolic volume, and enhanced left ventricular stroke volume [56, 57]. These mechanisms may provide a potential protective basis for obese individuals in T2MI, arising from oxygen supply–demand imbalance. Although the exact origins of fat and muscle mass are still unknown, patient BMI strongly correlates with fat area (r = 0.677, p < 0.001) and skeletal muscle area (r = 0.446, p < 0.001). Notably, the correlation between BMI and skeletal muscle area is significantly stronger in males than in females [58]. This disparity may contribute to women’s greater susceptibility to T2MI. (2) Obese patients frequently have concomitant metabolic syndrome (MetS). The prevalence of MetS is higher in women than in men [59]. Research shows that oxidative stress promotes pro-oxidative and pro-inflammatory responses in MetS patients. It does so by activating NF-κB-mediated inflammation [60], disrupting insulin signalling, and causing adipocyte dysfunction [61]. This increases the risk of cardiovascular events [62]. However, oxidative stress shows gender-specific differences. Elevated estrogen levels in women protect against MetS by upregulating endogenous antioxidant enzymes [63], binding to mitochondrial estrogen receptors to enhance antioxidant defenses, and inhibiting NADPH oxidase activity to reduce reactive oxygen species (ROS) production [64]. These mechanisms help shield women from MetS-related adverse effects [65]. Nevertheless, the decline in estrogen levels after menopause disrupts redox homeostasis. This disruption causes progressive accumulation of oxidative damage markers [66, 67], which promotes endothelial dysfunction and insulin resistance [48, 68]. Ultimately, these changes contribute to MetS development in older women and increase the risk of myocardial infarction.(3) Research indicates that females are at a higher risk of developing coronary microvascular dysfunction [69]. This increased risk is due to their higher levels of coronary microvascular resistance [70]. This condition can lead to a reduced coronary flow reserve, distal embolism, and an increased likelihood of experiencing the no-reflow phenomenon. This microvascular dysfunction can lead to myocardial hypoperfusion, which increases the risk of myocardial infarction. (4) Endothelial dysfunction is another major risk factor for cardiovascular disease, caused by an imbalance in the synthesis and availability of nitric oxide (NO) from the endothelium [71]. Inflammation significantly contributes to the development of endothelial dysfunction [72]. A study on sex differences in animals indicates that inflammatory markers, including tumor necrosis factor-α (TNF-α) and interleukin-6 (IL-6), are elevated in female mice [73]. Inflammatory mediators activate endothelial cells, which then become dysfunctional, reducing vasodilation and promoting a thrombotic state that raises the risk of cardiovascular events [72]. These findings suggest that females are more vulnerable to myocardial injury than males. Therefore, tailored prevention and management strategies for myocardial infarction are needed for this high-risk group.

Our research shows that among AIS patients aged 80 and older, normal-weight or underweight females have a significantly higher risk of T2MI than obese females. Conversely, in AIS patients under 80 years old, BMI did not significantly influence the occurrence of T2MI. The differences observed in this age subgroup are explained by the following factors. Previous studies indicate that aging causes significant structural changes in the heart, including alterations in geometry and increased risk of cardiac dysfunction in older patients [74, 75]. Cardiac output decreases with age, which affects overall heart function. In fact, research shows that early diastolic ventricular filling at age 70 is roughly 50% of that observed at age 30. Moreover, the workload on the heart, especially the left ventricle during ejection, increases gradually with age, leading to a decline in diastolic function in older adults over time [76].

Among AIS patients, normal weight individuals had a significantly higher risk of T2MI compared to obese patients, especially when the NIHSS score was 6 or higher. The NIHSS score has been linked to the severity of myocardial systolic dysfunction. A higher NIHSS score indicates a more pronounced disturbance in cardiac electrophysiological balance (OR 1.102; 95% CI 1.036–1.172; p < 0.001) [68]. This myocardial dysfunction leads to insufficient blood supply to the heart muscle, thereby increasing the risk of myocardial infarction. Moreover, individuals with higher NIHSS scores often have comorbidities like hypertension, diabetes, and coronary artery disease, each independently raising the risk of myocardial infarction [15].

A subgroup analysis of AIS patients showed that T2MI incidence was significantly higher in the normal weight group than the obese group, especially among those with impaired renal function, defined as an estimated glomerular filtration rate (eGFR) between 30 and 59 ml/min/1.73 m^2^. Additionally, T2MI incidence significantly increased with BUN levels from 6.9 to 20. A decrease in eGFR is significantly associated with a higher risk of cardiovascular events [77]. Furthermore, changes in eGFR are strongly linked to an increased risk of myocardial infarction in individuals with diabetes or prediabetes [78]. One possible explanation is that chronic kidney disease (CKD) can affect heart function by altering blood flow and causing the body to retain sodium and water. This may further raise the risk of myocardial infarction [79].

Additionally, the normal weight group had a significantly higher risk of T2MI than the obese group. This was true regardless of whether patients had atrial fibrillation or dysphagia. Diabetic patients in the low-weight group and non-diabetic individuals in the normal-weight group showed a higher risk of T2MI. This indicates that clinicians should pay special attention to underweight and normal-weight patients regarding their risk of T2MI.

## Limitations

Our investigation has some limitations. Firstly, the study was conducted at a single center. It primarily recruited patients from a regional population, which may limit the generalizability of the findings. Furthermore, the study may be affected by selection bias and unmeasured confounding factors. Further validation through multi-center, large-scale studies is needed. Secondly, BMI is a limited measure because it does not consider how fat is distributed in the body or the overall body composition of individuals. As a result, it does not help distinguish between muscle and fat tissue. Understanding how body composition differences affect the criteria distinguishing male and female bodies remains a challenge. Future animal cohort studies on body composition metrics should address this challenge. Thirdly, while BMI correlated with the incidence of T2MI after adjusting for various confounding factors, this study did not include data on pro-inflammatory indicators such as white blood cell count, C-reactive protein, and interleukin-6 (IL-6), or on anti-inflammatory factors like interleukin-4 (IL-4) and interleukin-10 (IL-10). Moreover, the intervention strategies, their duration, and assessments of inflammatory factor efficacy are insufficient to fully account for the impact of unmeasured confounders. Fourthly, diagnosing type 2 myocardial infarction (T2MI) objectively is challenging [80], as electrocardiograms and echocardiograms offer limited contributions to the diagnostic process [81]. While this study relied on the diagnostic expertise of three physicians from different specialties, inconsistencies in diagnoses may still exist, potentially influencing the conclusions drawn. Future research that relies on objective T2MI diagnoses is crucial to validate these findings.

## Conclusion

Our study confirmed that a sex-specific obesity paradox relates to the occurrence of T2MI in AIS patients. Therefore, in clinical practice, both sex and BMI should be comprehensively considered when assessing the risk of T2MI in AIS patients. This approach helps clinicians make more accurate decisions when evaluating patient risk.

## Supplementary Information

Supplementary Tables

## Data Availability

The data supporting the findings of this study are available from the Electronic Medical Record (EMR) system managed by our hospital. Access to this data is limited, as it was utilized under a license for the current study and, therefore, is not publicly accessible. However, the data are available from the corresponding author upon reasonable request.
