# Supplementary material for: Sex-specific obesity paradox and type 2 myocardial infarction in acute ischemic stroke (AIS) patients
Source: Biol Sex Differ. 2026 Jan 16;17:26. doi: 10.1186/s13293-026-00823-x (PMC12892716; doi:10.1186/s13293-026-00823-x)
Supplement: Supplementary file 1 — Supplementary Tables [file 13293_2026_823_MOESM1_ESM.docx]

Supplementary table 1 subjective and 0bjective Critera Used to the Diagnose Acute Myocardial Infarction following the fourth Uni.versal Definition of Myocardial Infarction Criteria

|  | **Type 2 Myocardial Infarction [n = 131, n(%)]** |
| --- | --- |
| Ischemic symptoms | 84(64.1) |
| Ischemic ECG changes | 42(32.1) |
| Pathologic Q-waves | 6(4.6) |
| New regional wall motion abnormalities | 6(4.6) |
| Culprit lesion on angiography | 8(6.1) |
| Objective myocardial infarction criteria (at least one) | 47(35.9) |

Supplementary table 2 baseline and clinical prognostic characteristics in male patients

| **Variables** | **Male  (n = 1891)** | **Underweight (n = 40)** | **Normal weight  (n = 432)** | **Overweight  (n = 671)** | **Obesity ( n = 748)** | ***P*-value** |
| --- | --- | --- | --- | --- | --- | --- |
| **Demographic data** |  |  |  |  |  |  |
| Age (Mean ± SD, years) | 67.73 ± 13.20 | 73.60 ± 11.44 | 70.05 ± 12.22 | 68.46 ± 12.69 | 65.42 ± 13.87 | <0.001 |
| BMI, (Mean ± SD, kg/m^2)^ | 24.57 ± 2.86 | 17.27 ± 1.07 | 21.37 ± 1.16 | 24.17 ± 0.52 | 27.17 ± 2.00 | <0.001 |
| **OCSP, n (%)** |  |  |  |  |  | 0.104 |
| TACI | 183 (9.68) | 4 (10.00) | 42 (9.72) | 67 (9.99) | 70 (9.36) |  |
| PACI | 1138 (60.18) | 31 (77.50) | 273 (63.19) | 379 (56.48) | 455 (60.83) |  |
| POCI | 499 (26.39) | 5 (12.50) | 98 (22.69) | 200 (29.81) | 196 (26.20) |  |
| LACI | 71 (3.75) | 0 (0.00) | 19 (4.40) | 25 (3.73) | 27 (3.61) |  |
| **TOAST, n (%)** |  |  |  |  |  | <0.001 |
| LAA | 778 (41.14) | 16 (40.00) | 157 (36.34) | 301 (44.86) | 304 (40.64) |  |
| CE | 191 (10.10) | 7 (17.50) | 40 (9.26) | 66 (9.84) | 78 (10.43) |  |
| SAA | 861 (45.53) | 12 (30.00) | 218 (50.46) | 295 (43.96) | 336 (44.92) |  |
| Other etiological types | 28 (1.48) | 3 (7.50) | 11 (2.55) | 5 (0.75) | 9 (1.20) |  |
| Unexplained | 33 (1.75) | 2 (5.00) | 6 (1.39) | 4 (0.60) | 21 (2.81) |  |
| **Drinking, n (%)** | 476 (25.17) | 6 (15.00) | 102 (23.61) | 149 (22.21) | 219 (29.28) | 0.006 |
| **Smoking, n (%)** |  |  |  |  |  | 0.117 |
| Never smoked | 1066 (56.37) | 15 (37.50) | 251 (58.10) | 375 (55.89) | 425 (56.82) |  |
| Previously smoked, now quit | 127 (6.72) | 4 (10.00) | 25 (5.79) | 55 (8.20) | 43 (5.75) |  |
| Currently still smoking | 698 (36.91) | 21 (52.50) | 156 (36.11) | 241 (35.92) | 280 (37.43) |  |
| **Past medical history, n (%)** |  |  |  |  |  |  |
| Cerebral hemorrhage | 55 (2.91) | 0 (0.00) | 14 (3.24) | 20 (2.98) | 21 (2.81) | 0.704 |
| Cerebral infarction | 374 (19.78) | 8 (20.00) | 77 (17.82) | 165 (24.59) | 124 (16.58) | 0.001 |
| Hypertension | 1316 (69.59) | 20 (50.00) | 263 (60.88) | 495 (73.77) | 538 (71.93) | <0.001 |
| Diabetes | 592 (31.31) | 7 (17.50) | 124 (28.70) | 231 (34.43) | 230 (30.75) | 0.045 |
| Myocardial infarction | 14 (0.74) | 0 (0.00) | 2 (0.46) | 4 (0.60) | 8 (1.07) | 0.642 |
| Atrial fibrillation | 206 (10.89) | 6 (15.00) | 41 (9.49) | 68 (10.13) | 91 (12.17) | 0.359 |
| Hyperlipoidemia | 10 (0.53) | 0 (0.00) | 3 (0.69) | 4 (0.60) | 3 (0.40) | 0.824 |
| Senile dementia | 13 (0.69) | 0 (0.00) | 1 (0.23) | 7 (1.04) | 5 (0.67) | 0.475 |
| Mental disorder | 5 (0.26) | 1 (2.50) | 1 (0.23) | 2 (0.30) | 1 (0.13) | 0.111 |
| COPD | 49 (2.59) | 5 (12.50) | 13 (3.01) | 14 (2.09) | 17 (2.27) | <0.001 |
| Spontaneous intracerebral hemorrhage | 27 (1.43) | 1 (2.50) | 4 (0.93) | 13 (1.94) | 9 (1.20) | 0.456 |
| Family history of stroke | 7 (0.37) | 0 (0.00) | 3 (0.69) | 1 (0.15) | 3 (0.40) | 0.48 |
| Transient ischemic attack | 14 (0.74) | 0 (0.00) | 3 (0.69) | 3 (0.45) | 8 (1.07) | 0.575 |
| Heart valve replacement surgery | 2 (0.11) | 0 (0.00) | 0 (0.00) | 0 (0.00) | 2 (0.27) | 0.376 |
| **Previous medication history, n (%)** |  |  |  |  |  |  |
| Antiplatelet therapy |  |  |  |  |  | 0.043 |
| Non-antiplatelet | 1630 (86.20) | 33 (82.50) | 388 (89.81) | 559 (83.31) | 650 (86.90) |  |
| Single drug antiplatelet | 236 (12.48) | 7 (17.50) | 41 (9.49) | 98 (14.61) | 90 (12.03) |  |
| Duplex antiplatelet agglutination therapy | 25 (1.32) | 0 (0.00) | 3 (0.69) | 14 (2.09) | 8 (1.07) |  |
| Anticoagulant medication | 25 (1.32) | 1 (2.50) | 5 (1.16) | 5 (0.75) | 14 (1.87) | 0.266 |
| Hypolipidemic drug therapy | 188 (9.94) | 5 (12.50) | 35 (8.10) | 71 (10.58) | 77 (10.29) | 0.506 |
| Blood pressure medication | 447 (23.64) | 4 (10.00) | 91 (21.06) | 175 (26.08) | 177 (23.66) | 0.048 |
| Hypoglycemic medication | 982 (51.93) | 16 (40.00) | 192 (44.44) | 374 (55.74) | 400 (53.48) | <0.001 |
| **Hospital medication, n (%)** |  |  |  |  |  |  |
| Antiplatelet therapy |  |  |  |  |  | <0.001 |
| Non-antiplatelet | 114 (6.03) | 5 (12.50) | 29 (6.71) | 38 (5.66) | 42 (5.61) |  |
| Single drug antiplatelet | 678 (35.85) | 27 (67.50) | 149 (34.49) | 256 (38.15) | 246 (32.89) |  |
| Duplex antiplatelet agglutination therapy | 1099 (58.12) | 8 (20.00) | 254 (58.80) | 377 (56.18) | 460 (61.50) |  |
| Anticoagulant medication | 48 (2.54) | 2 (5.00) | 12 (2.78) | 13 (1.94) | 21 (2.81) | 0.517 |
| Hypolipidemic drug therapy | 796 (42.09) | 10 (25.00) | 158 (36.57) | 286 (42.62) | 342 (45.72) | 0.003 |
| Blood pressure medication | 656 (34.69) | 6 (15.00) | 131 (30.32) | 253 (37.70) | 266 (35.56) | 0.004 |
| Hypoglycemic medication | 1432 (75.73) | 22 (55.00) | 311 (71.99) | 513 (76.45) | 586 (78.34) | 0.001 |
| **Cerebral vascular reperfusion therapy (CRT), n (%)** |  |  |  |  |  |  |
| Alteplase intravenous thrombolysis | 264 (13.96) | 10 (25.00) | 73 (16.90) | 69 (10.28) | 112 (14.97) | 0.002 |
| Arterial embolectomy | 27 (1.50) | 2 (5.56) | 7 (1.70) | 6 (0.95) | 12 (1.66) | 0.134 |
| **Severity of illness** |  |  |  |  |  |  |
| MRS score at admission, M (IQR) | 2 (1, 2) | 2 (1,3) | 2 (1,2) | 2 (1,2) | 1 (1,2) | <0.001 |
| NIHSS score at admission, M (IQR) | 2 (1, 5) | 3 (1.5,10) | 2 (1,4) | 2 (1,5) | 2 (1,5) | 0.038 |
| Dysphagia, n (%) | 209 (11.23) | 9 (22.50) | 44 (10.40) | 71 (10.76) | 85 (11.52) | 0.133 |
| MAP, (Mean ± SD, mmHg) | 106.43 ± 14.73 | 99.26 ± 13.65 | 104.13 ± 13.52 | 105.39 ± 13.35 | 109.06 ± 16.14 | <0.001 |
| Pulse, (Mean ± SD, Times/minute) | 77.27 ± 13.80 | 75.97 ± 14.01 | 75.44 ± 13.72 | 76.99 ± 13.77 | 78.64 ± 13.75 | 0.001 |
| **Laboratory parameters** |  |  |  |  |  |  |
| Cardiac troponin (cTn), [M (IQR), ng/mL] | 0.01 (0.01, 0.02) | 0.01 (0.01,0.02) | 0.01 (0.01,0.02) | 0.01 (0.01,0.02) | 0.01 (0.01,0.02) | 0.569 |
| Low-density lipoprotein, [M (IQR), mmol/L] | 2.59 (1.99, 3.24) | 2.24 (1.79,3.06) | 2.54 (1.94,3.13) | 2.57 (1.94,3.19) | 2.67 (2.08,3.30) | 0.011 |
| Homocysteine, [M (IQR), umol/L] | 15.80 (12.20, 21.90) | 14.15 (11.76,18.95) | 15.70 (12.10,20.96) | 15.80 (12.20,21.28) | 15.92 (12.40,22.98) | 0.316 |
| HbA1c, Mean ± SD | 6.71 ± 1.77 | 6.46 ± 1.87 | 6.61 ± 1.79 | 6.70 ± 1.75 | 6.78 ± 1.78 | 0.337 |
| Fasting blood glucose, (Mean ± SD, mmol/L) | 6.69 ± 2.70 | 6.15 ± 2.75 | 6.45 ± 2.53 | 6.79 ± 2.93 | 6.76 ± 2.56 | 0.093 |
| Serum creatinine, [M (IQR), umol/L] | 77.62 (67.00, 90.50) | 75.65 (65.78,85.45) | 76.20 (66.38,89.95) | 77.00 (66.78,89.85) | 78.85 (67.58,91.56) | 0.121 |
| Blood urea nitrogen, [M (IQR), mmol/L] | 5.20 (4.30, 6.40) | 5.70 (4.35,6.95) | 5.20 (4.20,6.45) | 5.20 (4.30,6.35) | 5.20 (4.30,6.40) | 0.625 |
| Uric acid, (Mean ± SD, umol/L) | 342.94 ± 100.83 | 333.82 ± 94.23 | 327.52 ± 98.23 | 338.23 ± 98.87 | 356.59 ± 102.84 | <0.001 |
| International normalized ratio,  Mean ± SD | 0.96 ± 0.12 | 0.97 ± 0.08 | 0.96 ± 0.11 | 0.96 ± 0.15 | 0.95 ± 0.10 | 0.347 |
| **clinical prognosis** |  |  |  |  |  |  |
| T2MI, n (%) | 70 (3.70) | 1 (2.50) | 22 (5.09) | 20 (2.98) | 27 (3.61) | 0.32 |
| Pneumonia, n (%) | 371 (19.62) | 14 (35.00) | 86 (19.91) | 132 (19.67) | 139 (18.58) | 0.088 |
| Died in hospital, n (%) | 27 (1.43) | 2 (5.00) | 4 (0.93) | 12 (1.79) | 9 (1.20) | 0.152 |
| Died in 90 days, n (%) | 55 (2.91) | 2 (5.00) | 11 (2.55) | 19 (2.83) | 23 (3.07) | 0.824 |
| Length of hospital stay, [M (IQR), days] | 10.(8, 13) | 10.5 (8,13) | 10 (8,13) | 10(8,13) | 10 (8,13) | 0.786 |
| NIHSS score at discharge, M (IQR) | 2 (1, 4) | 2.5 (0.25,7.75) | 2 (1,4) | 2 (1,4) | 2 (1,4) | 0.183 |
| MRS score at discharge, M (IQR) | 2 (1, 3) | 2 (1.00,3.75) | 1 (1,3) | 2 (1,3) | 2 (1,3) | 0.288 |
| Total hospitalization expenses, [M (IQR), ten thousand yuan] | 1.521(1.169, 2.065) | 1.624(1.353,2.219) | 1.52(1.148,2.071) | 1.461(1.135,1.975) | 1.557 (1.198,2.081) | 0.057 |
| Total cost of medication, [M (IQR), ten thousand yuan] | 0.713(0.449, 1.050) | 0.717(0.541,1.073) | 0.703(0.421,1.064) | 0.680(0.429,1.017) | 0.721(0.472,1.073) | 0.308 |
| Abbreviation: BMI, body mass index; OCSP, oxfordshire community stroke project; TACI, total anterior circulation infarcts; PACI, partial anterior circulation infarcts; POCI, posterior circulation infarcts; LACI, lacunar infarcts; TOAST, trial of org 10172 in acute stroke treatment; LAA, large-artery atherosclerosis; CE, cardioembolism; SAA, small-artery occlusion lacunar; COPD, chronic obstructive pulmonary disease; mRs, modified rankin scale; NIHSS, national institute of health stroke scale; MAP, mean arterial pressure; HbA1c, glycated hemoglobin; T2MI, type 2 myocardial infarction. | | | | | | |

Supplementary table 3 Baseline Characteristics Of AIS Patients With T2MI occurrence in female patients

| **Variables** | **Female  (n = 1064)** | **Non-T2MI  (n = 1003)** | **T2MI  (n = 61)** | ***P-value*** | **Univariate COX regression analysis** | |
| --- | --- | --- | --- | --- | --- | --- |
|  |  |  |  |  | **HR (95%CI)** | ***P-value*** |
| **Demographic data** |  |  |  |  |  |  |
| Age | 74.94 ± 12.33 | 74.49 ± 12.39 | 82.30 ± 8.50 | <0.001 | 1.07 (1.04 ~ 1.10) | <0.001 |
| **BMI** |  |  |  | <0.001 |  |  |
| Obesity | 370 (34.77) | 360 (35.89) | 10 (16.39) |  | 1.00 (Reference) |  |
| Underweight | 46 (4.32) | 40 (3.99) | 6 (9.84) |  | 5.15 (1.87 ~ 14.17) | 0.002 |
| Normal weight | 296 (27.82) | 268 (26.72) | 28 (45.90) |  | 3.59 (1.74 ~ 7.39) | <0.001 |
| Overweight | 352 (33.08) | 335 (33.40) | 17 (27.87) |  | 1.81 (0.83 ~ 3.95) | 0.137 |
| **OCSP** |  |  |  | 0.004 |  |  |
| TACI | 143 (13.44) | 128 (12.76) | 15 (24.59) |  | 1.00 (Reference) |  |
| PACI | 625 (58.74) | 586 (58.42) | 39 (63.93) |  | 0.59 (0.32 ~ 1.06) | 0.079 |
| POCI | 249 (23.40) | 242 (24.13) | 7 (11.48) |  | 0.26 (0.11 ~ 0.64) | 0.003 |
| LACI | 47 (4.42) | 47 (4.69) | 0 (0.00) |  | 0.00 (0.00 ~ Inf) | 0.995 |
| **TOAST** |  |  |  | <0.001 |  |  |
| LAA | 399 (37.50) | 382 (38.09) | 17 (27.87) |  | 1.00 (Reference) |  |
| CE | 184 (17.29) | 156 (15.55) | 28 (45.90) |  | 3.77 (2.06 ~ 6.89) | <0.001 |
| SAA | 450 (42.29) | 437 (43.57) | 13 (21.31) |  | 0.68 (0.33 ~ 1.39) | 0.289 |
| Other etiological types | 12 (1.13) | 11 (1.10) | 1 (1.64) |  | 1.96 (0.26 ~ 14.72) | 0.513 |
| Unexplained | 19 (1.79) | 17 (1.69) | 2 (3.28) |  | 2.58 (0.60 ~ 11.18) | 0.204 |
| **Drinking** | 22 (2.07) | 21 (2.09) | 1 (1.64) | 1 | 0.78 (0.11 ~ 5.60) | 0.801 |
| **Smoking** |  |  |  | 1 |  |  |
| Never smoked | 1043 (98.03) | 982 (97.91) | 61 (100.00) |  | 1.00 (Reference) |  |
| Previously smoked, now quit | 5 (0.47) | 5 (0.50) | 0 (0.00) |  | 0.00 (0.00 ~ Inf) | 0.998 |
| Currently still smoking | 16 (1.50) | 16 (1.60) | 0 (0.00) |  | 0.00 (0.00 ~ Inf) | 0.996 |
| **Past medical history** |  |  |  |  |  |  |
| Cerebral hemorrhage | 24 (2.26) | 24 (2.39) | 0 (0.00) | 0.437 | 0.00 (0.00 ~ Inf) | 0.995 |
| Cerebral infarction | 196 (18.42) | 181 (18.05) | 15 (24.59) | 0.2 | 1.45 (0.81 ~ 2.60) | 0.208 |
| Hypertension | 807 (75.85) | 759 (75.67) | 48 (78.69) | 0.593 | 1.18 (0.64 ~ 2.17) | 0.605 |
| Diabetes | 304 (28.57) | 288 (28.71) | 16 (26.23) | 0.677 | 0.88 (0.50 ~ 1.56) | 0.666 |
| Myocardial infarction | 2 (0.19) | 2 (0.20) | 0 (0.00) | 1 | 0.00 (0.00 ~ Inf) | 0.997 |
| Atrial fibrillation | 223 (20.96) | 196 (19.54) | 27 (44.26) | <0.001 | 3.11 (1.88 ~ 5.15) | <0.001 |
| Hyperlipoidemia | 6 (0.56) | 5 (0.50) | 1 (1.64) | 0.299 | 3.03 (0.42 ~ 21.89) | 0.271 |
| Senile dementia | 20 (1.88) | 19 (1.89) | 1 (1.64) | 1 | 0.87 (0.12 ~ 6.31) | 0.894 |
| Mental disorder | 7 (0.66) | 7 (0.70) | 0 (0.00) | 1 | 0.00 (0.00 ~ Inf) | 0.996 |
| COPD | 10 (0.94) | 10 (1.00) | 0 (0.00) | 1 | 0.00 (0.00 ~ Inf) | 0.995 |
| Spontaneous intracerebral hemorrhage | 12 (1.13) | 11 (1.10) | 1 (1.64) | 0.509 | 1.47 (0.20 ~ 10.62) | 0.702 |
| Family history of stroke | 5 (0.47) | 5 (0.50) | 0 (0.00) | 1 | 0.00 (0.00 ~ Inf) | 0.994 |
| Transient ischemic attack | 4 (0.38) | 3 (0.30) | 1 (1.64) | 0.211 | 5.09 (0.71 ~ 36.73) | 0.107 |
| Heart valve replacement surgery | 3 (0.28) | 3 (0.30) | 0 (0.00) | 1 | 0.00 (0.00 ~ Inf) | 0.996 |
| **Previous medication history** |  |  |  |  |  |  |
| Antiplatelet therapy |  |  |  | 0.316 |  |  |
| Non-antiplatelet | 931 (87.50) | 881 (87.84) | 50 (81.97) |  | 1.00 (Reference) |  |
| Single drug antiplatelet | 126 (11.84) | 116 (11.57) | 10 (16.39) |  | 1.50 (0.76 ~ 2.97) | 0.238 |
| Duplex antiplatelet agglutination therapy | 7 (0.66) | 6 (0.60) | 1 (1.64) |  | 2.75 (0.38 ~ 19.90) | 0.317 |
| Anticoagulant medication | 21 (1.97) | 18 (1.79) | 3 (4.92) | 0.219 | 2.72(0.85~8.67) | 0.092 |
| Hypolipidemic drug therapy | 99 (9.30) | 93 (9.27) | 6 (9.84) | 0.883 | 1.08 (0.46 ~ 2.50) | 0.862 |
| Blood pressure medication | 640 (60.15) | 601 (59.92) | 39 (63.93) | 0.534 | 1.18 (0.70 ~ 2.00) | 0.527 |
| Hypoglycemic medication | 241 (22.65) | 231 (23.03) | 10 (16.39) | 0.229 | 0.66 (0.34 ~ 1.31) | 0.234 |
| **Hospital medication** |  |  |  |  |  |  |
| Antiplatelet therapy |  |  |  | <0.001 |  |  |
| Non-antiplatelet | 96 (9.02) | 87 (8.67) | 9 (14.75) |  | 1.00 (Reference) |  |
| Single drug antiplatelet | 496 (46.62) | 457 (45.56) | 39 (63.93) |  | 0.83 (0.40 ~ 1.71) | 0.606 |
| Duplex antiplatelet agglutination therapy | 472 (44.36) | 459 (45.76) | 13 (21.31) |  | 0.28 (0.12 ~ 0.66) | 0.004 |
| Anticoagulant medication | 43 (4.04) | 40 (3.99) | 3 (4.92) | 0.981 | 1.26(0.39~4.02) | 0.696 |
| Hypolipidemic drug therapy | 479 (45.02) | 462 (46.06) | 17 (27.87) | 0.006 | 0.46 (0.26 ~ 0.81) | 0.007 |
| Blood pressure medication | 804 (75.56) | 771 (76.87) | 33 (54.10) | <0.001 | 0.37 (0.22 ~ 0.61) | <0.001 |
| Hypoglycemic medication | 332 (31.20) | 320 (31.90) | 12 (19.67) | 0.045 | 0.53 (0.28 ~ 1.00) | 0.05 |
| **Cerebral vascular reperfusion therapy (CRT)** |  |  |  |  |  |  |
| Alteplase intravenous thrombolysis | 183 (17.20) | 168 (16.75) | 15 (24.59) | 0.115 | 1.61 (0.90 ~ 2.88) | 0.111 |
| Arterial embolectomy | 21 (2.05) | 17 (1.77) | 4 (6.56) | 0.036 | 3.56 (1.29 ~ 9.82) | 0.014 |
| **Severity of illness** |  |  |  |  |  |  |
| MRS score at admission | 2 (1, 2) | 2(1, 2) | 1 (1, 3) | 0.44 | 1.07 (0.88 ~ 1.29) | 0.52 |
| NIHSS score at admission | 3 (1, 9) | 3 (1, 8) | 14 (7, 19) | <0.001 | 1.09 (1.07 ~ 1.12) | <0.001 |
| Dysphagia | 221 (20.97) | 182 (18.33) | 39 (63.93) | <0.001 | 7.14 (4.23 ~ 12.04) | <0.001 |
| MAP | 105.51 ± 14.24 | 105.66 ± 13.89 | 103.04 ± 19.03 | 0.294 | 0.99 (0.97 ~ 1.01) | 0.159 |
| Pulse | 79.97 ± 16.62 | 79.68 ± 16.58 | 84.72 ± 16.82 | 0.021 | 1.01 (1.01 ~ 1.03) | 0.021 |
| **Laboratory parameters** |  |  |  |  |  |  |
| Cardiac troponin (cTn) | 0.01 (0.01, 0.02) | 0.01 (0.01, 0.02) | 0.17 (0.05, 0.48) | <0.001 | 1.73 (1.53 ~ 1.96) | <0.001 |
| Low-density lipoprotein | 2.71 (2.11, 3.41) | 2.73 (2.12, 3.42) | 2.49 (2.07, 3.20) | 0.172 | 0.84 (0.64 ~ 1.10) | 0.198 |
| Homocysteine | 13.90 (10.50, 19.20) | 13.80 (10.40, 18.80) | 18.20 (12.40, 25.70) | <0.001 | 1.02 (1.01 ~ 1.04) | 0.006 |
| HbA1c | 6.60 ± 1.59 | 6.62 ± 1.59 | 6.39 ± 1.64 | 0.288 | 0.91 (0.75 ~ 1.09) | 0.291 |
| Fasting blood glucose | 6.81 ± 2.68 | 6.76 ± 2.63 | 7.62 ± 3.28 | 0.014 | 1.09 (1.02 ~ 1.17) | 0.014 |
| Serum creatinine | 64.14 (54.83, 79.03) | 63.90 (54.34, 77.28) | 79.10 (61.74, 101.00) | <0.001 | 1.01 (1.01 ~ 1.01) | <0.001 |
| Blood urea nitrogen | 5.00 (4.10, 6.30) | 5.00 (4.10, 6.20) | 6.70 (5.10, 8.40) | <0.001 | 1.21 (1.14 ~ 1.28) | <0.001 |
| Uric acid | 310.09 ± 106.69 | 307.00 ± 103.42 | 360.86 ± 142.41 | 0.005 | 1.01 (1.01 ~ 1.01) | <0.001 |
| International normalized ratio | 0.96 ± 0.16 | 0.96 ± 0.16 | 1.00 ± 0.11 | 0.072 | 2.06 (0.91 ~ 4.68) | 0.085 |
| **clinical prognosis** |  |  |  |  |  |  |
| Pneumonia, | 295 (27.73) | 252 (25.12) | 43 (70.49) | <0.001 | 6.53 (3.77 ~ 11.32) | <0.001 |
| Died in hospital | 30 (2.82) | 16 (1.60) | 14 (22.95) | <0.001 | 12.34 (6.78 ~ 22.45) | <0.001 |
| Died in 90 days | 52 (4.89) | 33 (3.29) | 19 (31.15) | <0.001 | 10.11 (5.88 ~ 17.41) | <0.001 |
| Length of hospital stay | 11.00 (9.00, 14.00) | 11.00 (9.00, 14.00) | 17.00 (11.00, 25.00) | <0.001 | 1.06 (1.04 ~ 1.08) | <0.001 |
| NIHSS score at discharge | 3 (1, 8) | 2 (1, 7) | 10.5 (5, 16) | <0.001 | 1.10 (1.07 ~ 1.12) | <0.001 |
| MRS score at discharge | 2 (1, 4) | 2 (1, 4) | 4(3, 5) | <0.001 | 1.79 (1.49 ~ 2.16) | <0.001 |
| Total hospitalization expenses | 1.602 (1.201, 2.227) | 1.576 (1.192, 2.169) | 25.815 (19.778, 40.227) | <0.001 | 1.01 (1.01 ~ 1.01) | <0.001 |
| Total cost of medication | 0.775 (0.481, 1.177) | 0.755 (0.469, 1.145) | 1.182 (0.789, 1.838) | <0.001 | 1.01 (1.01 ~ 1.01) | <0.001 |
| Abbreviation: HR, hazard ratio; CI, confidence interval; Abbreviation: BMI, body mass index; OCSP, oxfordshire community stroke project; TACI, total anterior circulation infarcts; PACI, partial anterior circulation infarcts; POCI, posterior circulation infarcts; LACI, lacunar infarcts; TOAST, trial of org 10172 in acute stroke treatment; LAA, large-artery atherosclerosis; CE, cardioembolism; SAA, small-artery occlusion lacunar; COPD, chronic obstructive pulmonary disease; mRs, modified rankin scale; NIHSS, national institute of health stroke scale; MAP, mean arterial pressure; HbA1c, glycated hemoglobin; T2MI, type 2 myocardial infarction. | | | | | | |

Supplementary table 4 Baseline Characteristics Of AIS Patients With T2MI occurrence in male patients

| **Variables** | **Male  (n = 1891)** | **Non-T2MI (n = 1821)** | **T2MI  (n = 70)** | ***P-value*** | **Univariate COX regression analysis** | |
| --- | --- | --- | --- | --- | --- | --- |
|  |  |  |  |  | **HR (95%CI)** | ***P*-value** |
| **Demographic data** |  |  |  |  |  |  |
| Age | 67.73 ± 13.20 | 67.49 ± 13.09 | 74.03 ± 14.48 | <0.001 | 1.04 (1.02 ~ 1.06) | <0.001 |
| **BMI** |  |  |  | 0.32 |  |  |
| Obesity | 748 (39.56) | 721 (39.59) | 27 (38.57) |  | 1.00 (Reference) |  |
| Underweight | 40 (2.12) | 39 (2.14) | 1 (1.43) |  | 0.69 (0.09 ~ 5.05) | 0.711 |
| Normal weight | 432 (22.85) | 410 (22.52) | 22 (31.43) |  | 1.42 (0.81 ~ 2.50) | 0.22 |
| Overweight | 671 (35.48) | 651 (35.75) | 20 (28.57) |  | 0.83 (0.46 ~ 1.47) | 0.519 |
| **OCSP** |  |  |  | 0.296 |  |  |
| TACI | 183 (9.68) | 175 (9.61) | 8 (11.43) |  | 1.00 (Reference) |  |
| PACI | 1138 (60.18) | 1092 (59.97) | 46 (65.71) |  | 0.93 (0.44 ~ 1.98) | 0.855 |
| POCI | 499 (26.39) | 487 (26.74) | 12 (17.14) |  | 0.55 (0.22 ~ 1.34) | 0.189 |
| LACI | 71 (3.75) | 67 (3.68) | 4 (5.71) |  | 1.31 (0.39 ~ 4.34) | 0.661 |
| **TOAST** |  |  |  | <0.001 |  |  |
| LAA | 778 (41.14) | 752 (41.30) | 26 (37.14) |  | 1.00 (Reference) |  |
| CE | 191 (10.10) | 167 (9.17) | 24 (34.29) |  | 3.91 (2.25 ~ 6.81) | <0.001 |
| SAA | 861 (45.53) | 842 (46.24) | 19 (27.14) |  | 0.66 (0.36 ~ 1.19) | 0.166 |
| Other etiological types | 28 (1.48) | 27 (1.48) | 1 (1.43) |  | 1.08 (0.15 ~ 7.94) | 0.942 |
| Unexplained | 33 (1.75) | 33 (1.81) | 0 (0.00) |  | 0.00 (0.00 ~ Inf) | 0.995 |
| **Drinking** | 476 (25.17) | 463 (25.43) | 13 (18.57) | 0.195 | 0.67 (0.37 ~ 1.23) | 0.199 |
| **Smoking** |  |  |  | 0.659 |  |  |
| Never smoked | 1066 (56.37) | 1027 (56.40) | 39 (55.71) |  | 1.00 (Reference) |  |
| Previously smoked, now quit | 127 (6.72) | 124 (6.81) | 3 (4.29) |  | 0.64 (0.20 ~ 2.07) | 0.454 |
| Currently still smoking | 698 (36.91) | 670 (36.79) | 28 (40.00) |  | 1.10 (0.68 ~ 1.79) | 0.699 |
| **Past medical history** |  |  |  |  |  |  |
| Cerebral hemorrhage | 55 (2.91) | 53 (2.91) | 2 (2.86) | 1 | 0.97 (0.24 ~ 3.98) | 0.971 |
| Cerebral infarction | 374 (19.78) | 356 (19.55) | 18 (25.71) | 0.204 | 1.41 (0.82 ~ 2.40) | 0.213 |
| Hypertension | 1316 (69.59) | 1257 (69.03) | 59 (84.29) | 0.006 | 2.37 (1.24 ~ 4.51) | 0.009 |
| Diabetes | 592 (31.31) | 567 (31.14) | 25 (35.71) | 0.418 | 1.22 (0.75 ~ 1.99) | 0.423 |
| Myocardial infarction | 14 (0.74) | 13 (0.71) | 1 (1.43) | 0.411 | 2.00 (0.28 ~ 14.38) | 0.492 |
| Atrial fibrillation | 206 (10.89) | 184 (10.10) | 22 (31.43) | <0.001 | 3.88 (2.34 ~ 6.42) | <0.001 |
| Hyperlipoidemia | 10 (0.53) | 9 (0.49) | 1 (1.43) | 0.315 | 2.75 (0.38 ~ 19.81) | 0.315 |
| Senile dementia | 13 (0.69) | 13 (0.71) | 0 (0.00) | 1 | 0.00 (0.00 ~ Inf) | 0.995 |
| Mental disorder | 5 (0.26) | 4 (0.22) | 1 (1.43) | 0.172 | 6.11 (0.85 ~ 43.96) | 0.072 |
| COPD | 49 (2.59) | 48 (2.64) | 1 (1.43) | 0.81 | 0.54 (0.07 ~ 3.87) | 0.537 |
| Spontaneous intracerebral hemorrhage | 27 (1.43) | 26 (1.43) | 1 (1.43) | 1 | 1.01 (0.14 ~ 7.25) | 0.995 |
| Family history of stroke | 7 (0.37) | 6 (0.33) | 1 (1.43) | 0.232 | 3.98 (0.55 ~ 28.66) | 0.17 |
| Transient ischemic attack | 14 (0.74) | 14 (0.77) | 0 (0.00) | 1 | 0.00 (0.00 ~ Inf) | 0.995 |
| Heart valve replacement surgery | 2 (0.11) | 2 (0.11) | 0 (0.00) | 1 | 0.00 (0.00 ~ Inf) | 0.996 |
| **Previous medication history** |  |  |  |  |  |  |
| Antiplatelet therapy |  |  |  | 0.313 |  |  |
| Non-antiplatelet | 1630 (86.20) | 1572 (86.33) | 58 (82.86) |  | 1.00 (Reference) |  |
| Single drug antiplatelet | 236 (12.48) | 224 (12.30) | 12 (17.14) |  | 1.43 (0.77 ~ 2.66) | 0.259 |
| Duplex antiplatelet agglutination therapy | 25 (1.32) | 25 (1.37) | 0 (0.00) |  | 0.00 (0.00 ~ Inf) | 0.994 |
| Anticoagulant medication | 25 (1.32) | 22 (1.21) | 3 (4.29) | 0.062 | 3.46(1.09~11.00) | 0.035 |
| Hypolipidemic drug therapy | 188 (9.94) | 176 (9.67) | 12 (17.14) | 0.04 | 1.58 (0.97 ~ 2.56) | 0.066 |
| Blood pressure medication | 982 (51.93) | 938 (51.51) | 44 (62.86) | 0.062 | 1.90 (1.02 ~ 3.53) | 0.043 |
| Hypoglycemic medication | 447 (23.64) | 428 (23.50) | 19 (27.14) | 0.482 | 1.20 (0.71 ~ 2.04) | 0.488 |
| **Hospital medication** |  |  |  |  |  |  |
| Antiplatelet therapy |  |  |  | <0.001 |  |  |
| Non-antiplatelet | 114 (6.03) | 100 (5.49) | 14 (20.00) |  | 1.00 (Reference) |  |
| Single drug antiplatelet | 678 (35.85) | 644 (35.37) | 34 (48.57) |  | 0.40 (0.21 ~ 0.74) | 0.004 |
| Duplex antiplatelet agglutination therapy | 1099 (58.12) | 1077 (59.14) | 22 (31.43) |  | 0.16 (0.08 ~ 0.31) | <0.001 |
| Anticoagulant medication | 48 (2.54) | 42 (2.31) | 6 (8.57) | 0.004 | 3.73(1.62~8.62) | 0.002 |
| Hypolipidemic drug therapy | 796 (42.09) | 773 (42.45) | 23 (32.86) | 0.111 | 0.67 (0.41 ~ 1.10) | 0.117 |
| Blood pressure medication | 1432 (75.73) | 1384 (76.00) | 48 (68.57) | 0.155 | 0.70 (0.42 ~ 1.16) | 0.163 |
| Hypoglycemic medication | 656 (34.69) | 636 (34.93) | 20 (28.57) | 0.273 | 0.75 (0.45 ~ 1.26) | 0.276 |
| **Cerebral vascular reperfusion therapy (CRT)** |  |  |  |  |  |  |
| Alteplase intravenous thrombolysis | 264 (13.96) | 254 (13.95) | 10 (14.29) | 0.936 | 1.03 (0.53 ~ 2.01) | 0.932 |
| Arterial embolectomy | 27 (1.50) | 25 (1.44) | 2 (2.99) | 0.611 | 2.06 (0.50 ~ 8.40) | 0.315 |
| **Severity of illness** |  |  |  |  |  |  |
| MRS score at admission | 2 (1, 2) | 2 (1, 2) | 2 (1, 3) | 0.005 | 1.39 (1.17 ~ 1.63) | <0.001 |
| NIHSS score at admission | 2 (1, 5) | 2 (1, 4) | 6 (2, 14) | <0.001 | 1.09 (1.07 ~ 1.12) | <0.001 |
| Dysphagia | 209 (11.23) | 177 (9.88) | 32 (46.38) | <0.001 | 7.10 (4.43 ~ 11.40) | <0.001 |
| MAP | 106.43 ± 14.73 | 106.56 ± 14.62 | 103.05 ± 17.32 | 0.051 | 0.98 (0.97 ~ 1.00) | 0.051 |
| Pulse | 77.27 ± 13.80 | 77.03 ± 13.57 | 83.31 ± 17.90 | 0.005 | 1.03 (1.01 ~ 1.04) | <0.001 |
| **Laboratory parameters** |  |  |  |  |  |  |
| Cardiac troponin (cTn) | 0.01 (0.01, 0.02) | 0.01 (0.01, 0.02) | 0.18 (0.08, 0.48) | <0.001 | 1.70 (1.52 ~ 1.92) | <0.001 |
| Low-density lipoprotein | 2.59 (1.99, 3.24) | 2.60 (2.00, 3.25) | 2.35 (1.65, 2.94) | 0.025 | 0.78 (0.60 ~ 1.02) | 0.072 |
| Homocysteine | 15.80 (12.20, 21.90) | 15.70 (12.20, 21.70) | 17.90 (13.40, 27.00) | 0.037 | 1.01 (1.00 ~ 1.03) | 0.098 |
| HbA1c | 6.71 ± 1.77 | 6.71 ± 1.77 | 6.64 ± 1.77 | 0.747 | 0.98 (0.85 ~ 1.12) | 0.747 |
| Fasting blood glucose | 6.69 ± 2.70 | 6.68 ± 2.69 | 7.00 ± 2.93 | 0.321 | 1.04 (0.96 ~ 1.13) | 0.318 |
| Serum creatinine | 77.62 (67.00, 90.50) | 77.10 (66.90, 89.60) | 101.44 (78.58, 134.49) | <0.001 | 1.01 (1.01 ~ 1.01) | <0.001 |
| Blood urea nitrogen | 5.20 (4.30, 6.40) | 5.20 (4.30, 6.30) | 6.90 (5.50, 10.70) | <0.001 | 1.22 (1.16 ~ 1.28) | <0.001 |
| Uric acid | 342.94 ± 100.83 | 342.05 ± 98.38 | 366.07 ± 150.37 | 0.189 | 1.01 (1.01 ~ 1.01) | 0.047 |
| International normalized ratio | 0.96 ± 0.12 | 0.95 ± 0.12 | 1.04 ± 0.18 | <.001 | 3.18 (2.00 ~ 5.05) | <0.001 |
| **clinical prognosis** |  |  |  |  |  |  |
| Pneumonia, | 371 (19.62) | 330 (18.12) | 41 (58.57) | <0.001 | 5.98 (3.72 ~ 9.62) | <0.001 |
| Died in hospital | 27 (1.43) | 18 (0.99) | 9 (12.86) | <0.001 | 11.23 (5.58 ~ 22.63) | <0.001 |
| Died in 90 days | 55 (2.91) | 42 (2.31) | 13 (18.57) | <0.001 | 8.15 (4.46 ~ 14.89) | <0.001 |
| Length of hospital stay | 10.00 (8.00, 13.00) | 10.00 (8.00, 13.00) | 14.50 (10.00, 21.00) | <0.001 | 1.06 (1.04 ~ 1.07) | <0.001 |
| NIHSS score at discharge | 2 (1, 4) | 2 (1, 4) | 10 (2, 16) | <0.001 | 1.12 (1.09 ~ 1.14) | <0.001 |
| MRS score at discharge | 2 (1, 3) | 1 (1, 3) | 4 (1, 5) | <0.001 | 1.75 (1.51 ~ 2.03) | <0.001 |
| Total hospitalization expenses | 1.521 (1.170, 2.065) | 1.507(1.162, 2.026) | 2.251(1.6950, 3.751) | <0.001 | 1.00 (1.00 ~ 1.00) | 0.132 |
| Total cost of medication | 0.713 (0.449, 1.050) | 0.703 (0.445, 1.036) | 0.995 (0.672, 1.716) | <0.001 | 1.01 (1.01 ~ 1.01) | <0.001 |
| Abbreviation: HR, hazard ratio; CI, confidence interval; Abbreviation: BMI, body mass index; OCSP, oxfordshire community stroke project; TACI, total anterior circulation infarcts; PACI, partial anterior circulation infarcts; POCI, posterior circulation infarcts; LACI, lacunar infarcts; TOAST, trial of org 10172 in acute stroke treatment; LAA, large-artery atherosclerosis; CE, cardioembolism; SAA, small-artery occlusion lacunar; COPD, chronic obstructive pulmonary disease; mRs, modified rankin scale; NIHSS, national institute of health stroke scale; MAP, mean arterial pressure; HbA1c, glycated hemoglobin; T2MI, type 2 myocardial infarction. | | | | | | |

Supplementary table 5 Univariate and multivariate Cox regression analysis of T2MI occurrence in female patients

| **Variables** | **Univariate analysis** | | **Multivariate analysis** | |
| --- | --- | --- | --- | --- |
|  | **HR (95%CI)** | **P-value** | **HR (95%CI)** | **P-value** |
| **Model 1** |  |  |  |  |
| BMI | 0.82 (0.76 ~ 0.89) | <0.001 | 0.85 (0.78 ~ 0.92) | <0.001 |
| Age | 1.07 (1.04 ~ 1.10) | <0.001 | 1.02 (0.99 ~ 1.06) | 0.116 |
| Atrial fibrillation | 3.11 (1.88 ~ 5.15) | <0.001 | 1.21 (0.68 ~ 2.17) | 0.515 |
| NIHSS score at admission | 1.09 (1.07 ~ 1.12) | <0.001 | 1.04 (1.01 ~ 1.07) | 0.047 |
| Dysphagia | 7.14 (4.23 ~ 12.04) | <0.001 | 3.01 (1.45 ~ 6.26) | 0.003 |
| Serum creatinine | 1.01 (1.01 ~ 1.01) | <0.001 | 1.00 (1.00 ~ 1.01) | 0.354 |
| Blood urea nitrogen | 1.21 (1.14 ~ 1.28) | <0.001 | 1.09 (1.01 ~ 1.19) | 0.044 |
| International normalized ratio | 2.06 (0.91 ~ 4.68) | 0.085 | 1.07 (0.32 ~ 3.61) | 0.909 |
| **Model 2** |  |  |  |  |
| BMI 4 groups |  |  |  |  |
| Obesity | 1.00 (Reference) |  | 1.00 (Reference) |  |
| Underweight | 5.15 (1.87 ~ 14.17) | 0.002 | 4.07 (1.45 ~ 11.44) | 0.008 |
| Normal weight | 3.59 (1.74 ~ 7.39) | <0.001 | 3.48 (1.65 ~ 7.35) | 0.001 |
| Overweight | 1.81 (0.83 ~ 3.95) | 0.137 | 2.12 (0.96 ~ 4.67) | 0.062 |
| Age | 1.07 (1.04 ~ 1.10) | <0.001 | 1.02 (0.99 ~ 1.06) | 0.113 |
| Atrial fibrillation | 3.11 (1.88 ~ 5.15) | <0.001 | 1.24 (0.69 ~ 2.22) | 0.475 |
| NIHSS score at admission | 1.09 (1.07 ~ 1.12) | <0.001 | 1.04 (1.01 ~ 1.07) | 0.048 |
| Dysphagia | 7.14 (4.23 ~ 12.04) | <0.001 | 3.15 (1.52 ~ 6.52) | 0.002 |
| Serum creatinine | 1.01 (1.01 ~ 1.01) | <0.001 | 1.00 (1.00 ~ 1.01) | 0.178 |
| Blood urea nitrogen | 1.21 (1.14 ~ 1.28) | <0.001 | 1.08 (0.99 ~ 1.18) | 0.096 |
| International normalized ratio | 2.06 (0.91 ~ 4.68) | 0.085 | 1.13 (0.32 ~ 3.98) | 0.851 |
| Abbreviation: HR, hazard ratio; CI, confidence interval; BMI, body mass index. | | | | |

Supplementary table 6 Univariate and multivariate Cox regression analysis of T2M occurrence in male patients

| **Variables** | **Univariate analysis** | | **Multivariate analysis** | |
| --- | --- | --- | --- | --- |
|  | **HR (95%CI)** | **P-value** | **HR (95%CI)** | **P-value** |
| **Model 1** |  |  |  |  |
| BMI | 0.96 (0.88 ~ 1.04) | 0.301 | 0.97 (0.90 ~ 1.06) | 0.542 |
| Age | 1.04 (1.02 ~ 1.06) | <0.001 | 1.00 (0.98 ~ 1.03) | 0.702 |
| Atrial fibrillation | 3.88 (2.34 ~ 6.42) | <0.001 | 1.69 (0.95 ~ 3.02) | 0.076 |
| NIHSS score at admission | 1.09 (1.07 ~ 1.12) | <0.001 | 1.03 (0.99 ~ 1.07) | 0.097 |
| Dysphagia | 7.10 (4.43 ~ 11.40) | <0.001 | 2.77 (1.35 ~ 5.66) | 0.005 |
| Serum creatinine | 1.01 (1.01 ~ 1.01) | <0.001 | 1.00 (1.00 ~ 1.00) | 0.579 |
| Blood urea nitrogen | 1.22 (1.16 ~ 1.28) | <0.001 | 1.12 (1.04 ~ 1.21) | 0.003 |
| International normalized ratio | 3.18 (2.00 ~ 5.05) | <0.001 | 2.41 (1.13 ~ 5.15) | 0.023 |
| **Model 2** |  |  |  |  |
| BMI 4 groups |  |  |  |  |
| Obesity | 1.00 (Reference) |  | 1.00 (Reference) |  |
| Underweight | 0.69 (0.09 ~ 5.05) | 0.711 | 0.46 (0.06 ~ 3.41) | 0.447 |
| Normal weight | 1.42 (0.81 ~ 2.50) | 0.22 | 1.52 (0.86 ~ 2.71) | 0.151 |
| Overweight | 0.83 (0.46 ~ 1.47) | 0.519 | 0.83 (0.46 ~ 1.51) | 0.542 |
| Age | 1.04 (1.02 ~ 1.06) | <0.001 | 1.00 (0.98 ~ 1.03) | 0.728 |
| Atrial fibrillation | 3.88 (2.34 ~ 6.42) | <0.001 | 1.69 (0.94 ~ 3.03) | 0.078 |
| NIHSS score at admission | 1.09 (1.07 ~ 1.12) | <0.001 | 1.03 (1.00 ~ 1.07) | 0.078 |
| Dysphagia | 7.10 (4.43 ~ 11.40) | <0.001 | 2.76 (1.35 ~ 5.65) | 0.005 |
| Serum creatinine | 1.01 (1.01 ~ 1.01) | <0.001 | 1.00 (1.00 ~ 1.00) | 0.701 |
| Blood urea nitrogen | 1.22 (1.16 ~ 1.28) | <0.001 | 1.13 (1.04 ~ 1.22) | 0.004 |
| International normalized ratio | 3.18 (2.00 ~ 5.05) | <0.001 | 2.34 (1.01 ~ 5.43) | 0.049 |
| Abbreviation: HR, hazard ratio; CI, confidence interval; BMI, body mass index. | | | | |
